# Safety and Immunogenicity of H1/IC31®, an Adjuvanted TB Subunit Vaccine, in HIV-Infected Adults with CD4+ Lymphocyte Counts Greater than 350 cells/mm^3^: A Phase II, Multi-Centre, Double-Blind, Randomized, Placebo-Controlled Trial

**DOI:** 10.1371/journal.pone.0114602

**Published:** 2014-12-09

**Authors:** Klaus Reither, Lynn Katsoulis, Trevor Beattie, Nicolene Gardiner, Nicole Lenz, Khadija Said, Elirehema Mfinanga, Christian Pohl, Katherine L. Fielding, Hannah Jeffery, Benjamin M. Kagina, Elisabeth J. Hughes, Thomas J. Scriba, Willem A. Hanekom, Søren T. Hoff, Peter Bang, Ingrid Kromann, Claudia Daubenberger, Peter Andersen, Gavin J. Churchyard

**Affiliations:** 1 Swiss Tropical and Public Health Institute, Basel, Switzerland; 2 University Basel, Basel, Switzerland; 3 Ifakara Health Institute, Bagamoyo, Tanzania; 4 Aurum Institute, Johannesburg, South Africa; 5 London School of Hygiene and Tropical Medicine, London, United Kingdom; 6 South African Tuberculosis Vaccine Initiative, Institute of Infectious Disease and Molecular Medicine and School of Child and Adolescent Health, University of Cape Town, Cape Town, South Africa; 7 Statens Serum Institute, Department of Infectious Disease Immunology, Copenhagen, Denmark; 8 Statens Serum Institute, Department of Vaccine Development, Copenhagen, Denmark; 9 School of Public Health, University of Witwatersrand, Johannesburg, South Africa; Rush University, United States of America

## Abstract

**Background:**

Novel tuberculosis vaccines should be safe, immunogenic, and effective in various population groups, including HIV-infected individuals. In this phase II multi-centre, double-blind, placebo-controlled trial, the safety and immunogenicity of the novel H1/IC31 vaccine, a fusion protein of Ag85B-ESAT-6 (H1) formulated with the adjuvant IC31, was evaluated in HIV-infected adults.

**Methods:**

HIV-infected adults with CD4+ T cell counts >350/mm^3^ and without evidence of active tuberculosis were enrolled and followed until day 182. H1/IC31 vaccine or placebo was randomly allocated in a 5∶1 ratio. The vaccine was administered intramuscularly at day 0 and 56. Safety assessment was based on medical history, clinical examinations, and blood and urine testing. Immunogenicity was determined by a short-term whole blood intracellular cytokine staining assay.

**Results:**

47 of the 48 randomised participants completed both vaccinations. In total, 459 mild or moderate and 2 severe adverse events were reported. There were three serious adverse events in two vaccinees classified as not related to the investigational product. Local injection site reactions were more common in H1/IC31 versus placebo recipients (65.0% vs. 12.5%, p = 0.015). Solicited systemic and unsolicited adverse events were similar by study arm. The baseline CD4+ T cell count and HIV viral load were similar by study arm and remained constant over time. The H1/IC31 vaccine induced a persistent Th1-immune response with predominately TNF-α and IL-2 co-expressing CD4+ T cells, as well as polyfunctional IFN-γ, TNF-α and IL-2 expressing CD4+ T cells.

**Conclusion:**

H1/IC31 was well tolerated and safe in HIV-infected adults with a CD4+ Lymphocyte count greater than 350 cells/mm^3^. The vaccine did not have an effect on CD4+ T cell count or HIV-1 viral load. H1/IC31 induced a specific and durable Th1 immune response.

**Trial registration:**

Pan African Clinical Trials Registry (PACTR) PACTR201105000289276

## Introduction

Tuberculosis (TB) remains a global public health problem. One third of humankind is infected with *Mycobacterium tuberculosis (M.tb),* which according to the World Health Organization (WHO) led to almost 8.6 million new active TB cases and 1.3 million TB deaths in 2012 [1]. *Mycobacterium bovis* Bacille Calmette-Guérin (BCG), the only currently licensed TB vaccine, is effective in preventing severe progressive disease in children but has limited impact on adult pulmonary TB, the driving force of the TB global pandemic [2] [3]. Consequently, there is an urgent need to develop safe and effective TB vaccines to accelerate progress towards TB elimination.

Vaccination campaigns may administer TB vaccines to persons without knowing their HIV status. It is therefore essential to evaluate the safety and immunogenicity of TB vaccines in HIV-infected persons. Furthermore, HIV-infected individuals have an increased risk of developing active TB, which may be decreased by an effective TB vaccine that reduces the risk of reactivation of latent TB infection (LTBI) or prevents TB infection or reinfection.

The Statens Serum Institut Hybrid (H1) recombinant fusion protein (antigen (Ag)85B- Early Secretory Antigenic Target (ESAT)-6) TB vaccine, adjuvanted with IC31, has been shown to be safe and immunogenic in BCG unvaccinated, TB uninfected participants and in BCG vaccinated and latently TB infected participants [4] In this phase II trial we evaluated the safety and immunogenicity of H1/IC31administered to HIV-infected adults with CD4+ lymphocyte counts greater than 350 cells/mm^3^ and without evidence of active TB disease (PACTR Identifier: PACTR201105000289276).

## Methods

### Regulatory approval

The study was conducted in accordance with the Helsinki Declaration and Good Clinical Practice (GCP) and approved by the following local and national ethics committees and regulatory authorities: Medical Research Coordinating Committee of Tanzania, Institutional Review Board of the Ifakara Health Institute, Tanzanian Food and Drug Authority, South African Medicines Control Council and the Human Research Ethics Committee, University of Witwatersrand.

### Study design and sites

This was a phase II, multicentre, double-blind, randomized, placebo-controlled trial. Participants were eligible if they were between 18 and 55 years of age, HIV infected with CD4+ lymphocyte counts greater than 350/mm^3^, antiretroviral therapy naïve, generally healthy, had no evidence of active TB, had no history of receiving immunosuppressive medication, immunoglobulins, blood products or known hypersensitivity to any of the vaccine components. Women of child bearing potential were eligible if pregnancy was excluded and they agreed to use at least two forms of acceptable contraception from 21 days prior to administration of the study vaccine through to the end of the study.

The trial took place at two African research facilities, the rural Bagamoyo Site of the Ifakara Health Institute in Tanzania and the urban Tembisa Site of the Aurum Institute in South Africa. It was carried out between 19th of December 2011 (first participant first visit) and 10^th^ of September 2012 (last participant last visit); the first participant last visit was on the 2^nd^ of July 2012. Written informed consent was obtained from all literate patients. In case of illiteracy, informed oral consent was attested by an impartial witness and documented with the patient’s fingerprint according to ICH GCP guidelines (E6 (R1); 4.8.9), as approved by the above mentioned ethics committees and regulatory authorities. The protocol for this trial and CONSORT checklist are available as supporting information; see S1 Protocol and S1 Checklist.

### Randomisation and blinding

Participants were randomly allocated in a 5∶1 ratio to receive either H1/IC31 vaccine or placebo according to a computer-generated randomisation list. The pharmacist prepared the vaccination according the pre-prepared study randomisation list. The unequal randomisation was employed to maximise exposure to the H1/IC31 vaccine thereby increasing the chance of detecting a serious adverse event to the vaccine. The study monitors, investigators, and participants were blinded to study product. The study pharmacists prepared the investigational product. Syringes were masked with red tape in order to conceal a slight difference in the appearance of the H1/IC31 and placebo.

### Investigational product and vaccination

The Hybrid 1 (H1) vaccine is a recombinant fusion protein of the antigens Ag85B and ESAT-6 (Ag85B–ESAT-6), developed and manufactured by the Statens Serum Institut (Denmark). The adjuvant IC31 was developed by Intercell AG (Austria) and consists the cationic polyaminoacid KLK, which is composed of the amino acids lysine (K) and leucine (L), and ODN1a, a single stranded oligodeoxynucleotide with alternating sequences of the nucleic acids inosine and cytidine. A volume of 0.5 mL was administered providing a dose of 50 µg Ag85B-ESAT6 (antigen) and 500 nmol KLK +20 nmol ODN1a (adjuvant) or 0.5 mL Tris buffer (placebo).

The H1/IC31 vaccine or placebo was administered intramuscularly with a 22 to 25-gauge needle at study day 0 and 56, after confirming eligibility criteria. The first injection was given into the right and the second into the left deltoid muscle.

### Safety

All solicited local (pain, tenderness, erythema, induration, nodules) and systemic adverse events (AEs) (malaise, myalgia, headache, nausea, vomiting, athralgia, fatigue, chills, fever) and unsolicited AEs were coded by the Aurum Institute according to the MedDRA Dictionary Version 15.1. AEs and laboratory measures of safety were graded using the Division of AIDS Table for Grading the Severity of Adult and Pediatric Adverse Events (version 1.0, 2004) as mild, moderate, severe or potentially life-threatening and assessed for their relationship to study product. CD4+ T cell count and HIV-1 viral load were measured pre and 14 days post vaccination and at the study end.

### IFN-γ release assay

QuantiFERON-TB Gold In-Tube assay (QFT) (Celestis) was performed at screening and at day 182 to identify whether H1/IC31 induced a false positive result. The assay was carried out according to manufacturer’s instructions with a cut-off value of 0.35 IU IFN-γ/mL to either antigen (ESAT-6/CFP-10).

### Immunological assays

All immunological assays described below were performed by the South African TB Vaccine Initiative (SATVI) laboratory in Cape Town, South Africa.

***The whole blood intracellular cytokine staining assay*** was used to quantify antigen specific CD4+ and CD8+ T cell responses (interferon-gamma (IFN-γ), tumour necrosis factor-alpha (TNF-α), interleukin (IL)-2 and IL-17) after stimulation with peptide pools of Ag85B and ESAT-6 peptides (each consisting of 15-mer peptides, overlapping by 10 amino acids, final concentration of 2 µg/mL, GenScript) and recombinant H1 fusion protein (final concentration of 5 µg/mL, Satens Serum Institute). CD4+ and CD8+ T cell expression of IFN-γ, TNF-α and IL-2 was measured to assess the polyfunctional characteristics of H1/IC31 induced T cells. The primary immunogenicity endpoint was assessed at day 70 (14 days post 2nd vaccination).

Processing of whole blood started within 75 minutes after phlebotomy. One mL whole blood from each study participant and time point were either left unstimulated (negative control) or stimulated with peptide pools of Ag85B, ESAT-6, the H1 fusion protein and phytohemagglutinin (PHA) (positive controls). The co-stimulatory antibodies anti-CD28 and anti-CD49d (BD Biosciences, at 0.5 µg/mL each) were included in all assay conditions. The whole blood was incubated at 37°C for 7 hours and Brefeldin-A (Sigma-Aldrich, 10 µg/mL) was added thereafter. The blood sample was transferred to a waterbath at 37°C and incubated for a further 5 hours. Thereafter the waterbath was switched off and allowed to reach room temperature. Within the following 10 hours the blood was harvested with EDTA (Sigma-Aldrich, at 1.8 mM), red blood cells were lysed and white cells fixed with 9 ml of FACS lysing solution (BD Biosciences). Thereafter, white cells were pelleted and cryopreserved in cryosolution containing 50% RPMI (Lonza), 40% fetal calf serum (BioWest) and 10% DMSO (Sigma-Aldrich). The specimens were shipped to SATVI ensuring that the cold chain was maintained.

The subsequent intracellular cytokine staining (ICS) procedure was performed as follows: the stimulated, fixed and frozen white cells from whole blood were thawed in a water bath at 37°C. The thawed cells were transferred to labelled tubes containing phosphate buffered saline (PBS, BioWhittaker) and washed. Next, the cells were permeabilised using Perm/Wash solution (BD Biosciences). Cells were then stained with the following anti-human antibodies: CD3 BV421 (BD Biosciences, MOPC-21), CD4-QDot605 (Invitrogen, S3.5), CD8-Cy5.5PerCP (BD Biosciences, SK1), CCR7-PE (eBioscience, 150503), IFN-γ-Alexa 700 (BD Biosciences, B27), TNF-α-Cy7PE (eBioscience, Mb11), IL-2-FITC (BD Biosciences, 5344.111), IL-17-Alexa 647 (eBioscience, SCPL1362) and CD45RA BV 570 (eBioscience, H1100). Results of CCR7 and CD45RA staining are not presented here. Samples were stained, acquired and analysed in batches. For every ICS assay experiment, compensation controls (single stained positive and negative mouse kappa compensation beads) were included. These controls were processed in parallel with the study samples during the staining and acquisition process to allow post acquisition flow cytometry data compensation. After the samples were acquired on the BD LSRII cytometer (BD Biosciences, San Jose, CA), flow analysis was performed using FlowJo software (TreeStar version 9.6.2).

***Anti-Ag85B-ESAT-6 specific IgG antibody assay*** was used to measure antibodies specific to antigens of H1/IC31 as an indicator of humoral reactivity. Plasma was collected and frozen from ex vivo whole blood.

#### IFN-γ EliSpot assay

Prior to the start of the study both sites laboratories were trained on specimen processing and storage. Both sites passed their proficiency tests for PBMCs cell viability and number and were IATA certified for shipping specimens. IFN-γ EliSpot assay was not possible to conduct on PBMCs collected from the Tembisa site because the cell recovery upon thaw was too low (less than 100,000 cells). The cell recovery and yield was excellent for PBMCs from Bagamoyo. However, on Elispot assay, these PBMCs showed high level of spot forming units in the negative control wells that was indistinguishable from the response detected with the assay’s positive control (stimulation with PHA). Therefore, we could not reliably report the IFN-γ EliSpot assay results.

### Statistical considerations

A sample size of 40 vaccine and 8 placebo recipients was adequate to assess the safety and immunogenicity of the vaccine. Assuming an incidence of adverse events in the vaccine and placebo arm of 10 and 15 per 100 person years, the probability of observing at least one adverse event was 0.87 and 0.95, respectively. The sample size for immunogenicity response rates were based on the phase I study results where 100% responded by the 2nd vaccination. The sample size was adequate to show an immune response in 70–90% of vaccine recipients and an absolute difference of 66% in the proportion of vaccine and placebo recipients with an immune response with 90% power.

Laboratory and immunogenicity parameters were summarised by study arm and visit. The baseline is defined as the measurement at day 0. Comparing cytokine responses (magnitude of response using all participants) between study arms was conducted using the Wilcoxon rank-sum test and between time points using the Wilcoxon signed-ranks test. The CD4 and log10(HIV-1 viral load) change over follow-up was estimated for each individual using linear regression and the estimated slopes compared by study arm using the Wilcoxon rank-sum test. All AEs were summarised by system organ class and preferred term by treatment group and visit. The statistical analysis was performed using software STATA version 13.0.

The study protocol and the CONSORT checklist are available as supporting information.

## Results

### Study population

**Figure 1:**
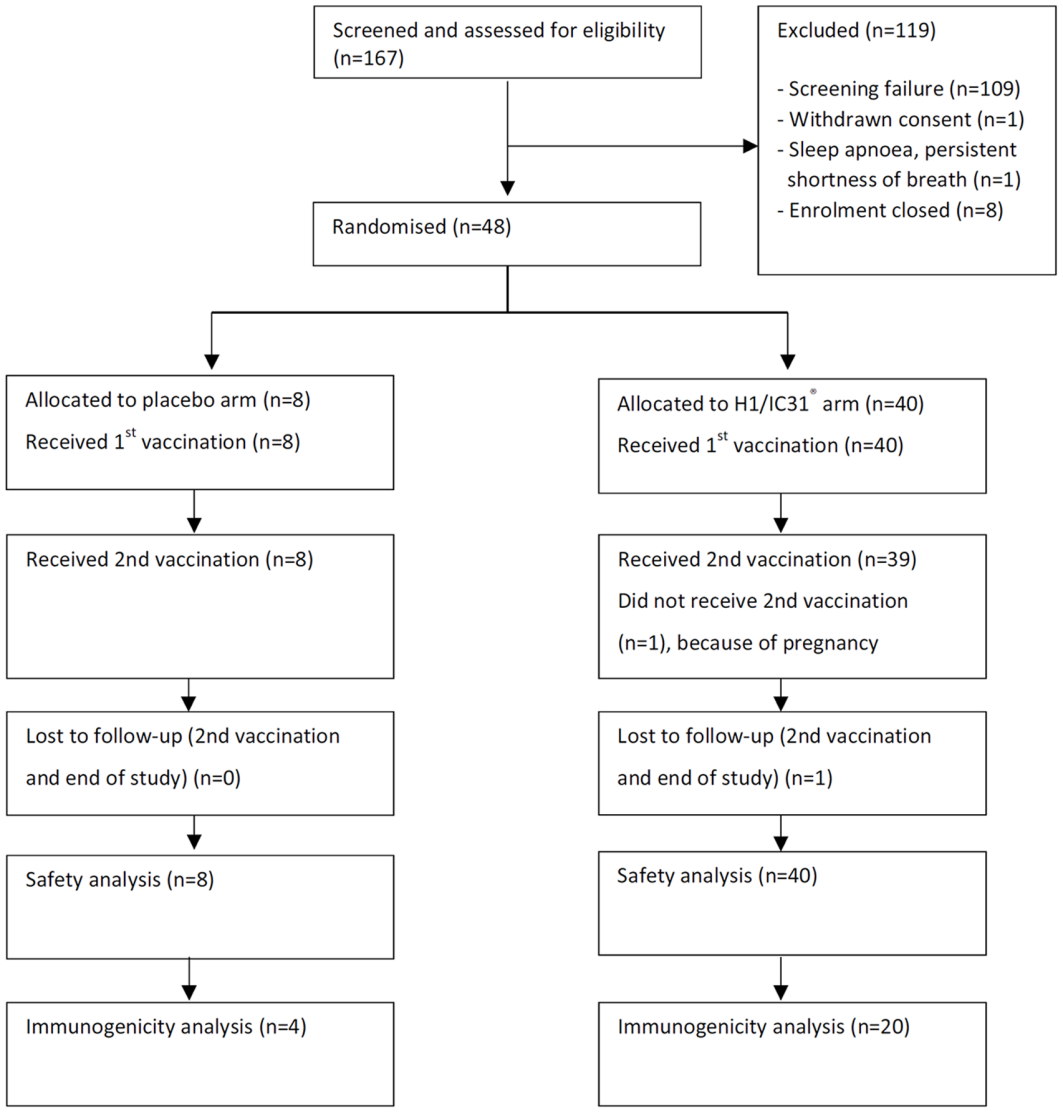
CONSORT diagram of subjects for screening, exclusions, randomisation, vaccination and follow-up in the vaccine trial.

In total, 167 adults were screened and 48 volunteers were enrolled (24 in Tanzania and South Africa respectively); 47 participants completed both vaccinations. The CONSORT diagram is shown in Fig. 1. Participants in the H1/IC31, versus placebo, arm were older (median age 35 vs 29.5 years, respectively) and less likely to be female (52.5% vs 75%, respectively) (Table 1). The overall proportion of self-reported BCG vaccination was 81.3%, similar by study arm. The median CD4+ T cell count, viral load and other baseline characteristics were similar by study arm (Table 1).

**Table 1:** Demographic and baseline characteristics of the study participants by vaccination group.

Variable		Placebo	H1/IC31
		(n = 8)	(n = 40)
Age	Median (IQR)	29.5 (28–41)	35 (30.5–41.5)
Gender	Female n (%)	6 (75.0)	21 (52.5)
Ethnic group*	Black n (%)	8 (100)	40 (100)
Body Mass Index	Median (IQR)	23.1 (20.2–27.0)	23.8 (20.3–30.8)
BCG vaccination (self-report)	Yes n (%)	6 (75.0)	33 (82.5)
CD4# (cells/uL)	Median (IQR)	556.5 (464–708)	620.5 (475–725)
Viral load○ (cp/mL)	Median (IQR)	17887 (544, 43297)	16968 (2228, 42547)

^*^other groups included Indian, White, Asian or mixed heritage.

# based on arithmetic mean of participant data from the two screening visits and day 0.

○ based on arithmetic mean of participant data from the first screening visit and day 0.

IQR interquartile range.

### Safety and reactogenicity

The safety analysis included data from both study sites. In total 461 AEs were reported (Table 2).

**Table 2:** Adverse events (AEs) during trial period (day 0–182) by vaccination group.

	Placebo		H1/IC31	
	n = 8		n = 40	
	All grades	Grade ≥3	All grades	Grade ≥3
**Solicited local AEs**				
*** Any***	**1**	**0**	**62**	**0**
Pain	0	0	21	0
Tenderness	0	0	10	0
Erythema	0	0	9	0
Induration	1	0	20	0
Nodules	0	0	2	0
**Solicited systemic AEs**				
*** Any***	**17**	**0**	**84**	**0**
Malaise	0	0	2	0
Myalgia	4	0	15	0
Headache	4	0	23	0
Nausea	2	0	0	0
Vomiting	0	0	1	0
Arthralgia	1	0	15	0
Fatigue	3	0	22	0
Chills	2	0	2	0
Fever	1	0	4	0
**Unsolicited AEs**				
*** Any***	**46**	**0**	**251**	**2** *
Blood/lymphatic disorders	12	0	48	0
Infections and infestations	10	0	44	0
Renal/urinary disorders	2	0	28	0
Other AEs	22	0	131	2*
**TOTAL**	**64**	**0**	**397**	**2** *

*reported as possibly related.

H1/IC31 versus placebo recipients were more likely to have one or more local injection site AE (H1/IC31∶26/40 (65.0%), placebo: 1/8 (12.5%), p = 0.015). The local AEs in the H1/IC31 arm were graded as mild (55/62, 88.7%) or moderate (7/62, 11.3%) and were either not related (1/62, 1.6%), possibly (23/62, 37.1%), probably (37/62, 59.7%) or (1/62, 1.6%) definitely related. In the H1/IC31 arm, the most frequent solicited local AEs were pain (21/62, 33.9%) and induration (20/62, 32.3%). The one local AE in the placebo arm was assessed as mild and probably related.

29/40 (72.5%) of H1/IC31 participants experienced a total of 84 solicited systemic AEs, which were graded as mild (72/84, 85.7%) or moderate (12/84, 14.3%) and 51.2% (43/84) and 22.6% (19/84) were assessed as possibly or probably related respectively. In the placebo arm, 7/8 (87.5%) participants experienced a total of 17 systemic AEs. These were graded as mild (17/17, 100%) and either unrelated (4/17, 23.5%) or possibly related (13/17, 76.5%). In the H1/IC31 and placebo arms, headache (23/84, 27.4%; 4/17, 23.5%) and fatigue (22/84, 26.2%; 3/17, 17.6%) were the most common solicited systemic AEs. The percentage of patients with at least one solicited systemic AE was similar by study arm (H1/IC31 29/40 [72.5%] vs placebo 7/8 [87.5%]; p = 0.4).

All participants reported one or more unsolicited systemic AE (297 in total). The 40 H1/IC31 recipients experienced 251 unsolicited AEs, which were graded as either mild (215/251, 85.7%), moderate (33/251, 13.2%) of severe (2/251, 0.8%), and one AE was ungraded (pregnancy). Of the 251 unsolicited AEs among H1/IC31 recipients, 152 (60.6%) were not related, 96 (38.3%) were possibly related and 3 (1.2%) were probably related. 46 unsolicited AEs were reported in the placebo group, all considered unrelated (34/46, 73.9%) or possibly related (12/46, 26.1%) and graded as either mild (40/46, 87%) or moderate (6/46, 13%). There were three serious individual case safety reports from two H1/IC31 participants, all classified as unrelated to the investigational product. One participant had two separate hospitalisations for malaria and drainage of a perianal abscess. The second participant delivered a premature baby that died as a result of respiratory failure. The participant had a history of one miscarriage in the last trimester and one delivery of another child, who died soon after birth. In the H1/IC31 and placebo arms, unsolicited systemic AE were most frequently categorised as blood and lymphatic disorders (48/251, 19.1%; 12/46, 26.1%) or infections and infestations (44/251, 17.5%; 10/46, 21.7%).

The frequencies of one or more local and systemic solicited AE per participant was similar after the first and the second vaccination with H1/IC31 (solicited local: 50% vs 32.5%, p = 0.18; solicited systemic: 60% vs 50%, p = 0.25), while unsolicited AEs were less frequent after the second H1/IC31 vaccination (97.5% vs 80%, p = 0.007).

QFT positive, versus negative, H1/IC31 recipients had more solicited local reactions (82.3% vs 47.8%, p = 0.046), but not solicited systemic (82.3% vs 65.2%, p = 0.3) or unsolicited AEs (100% vs 100%).

There were no significant differences in haematology, biochemistry or urinalysis test results at any visit by study arm. The median CD4 slope (per day) over the 182 day follow-up period was −0.86 and −0.59 cells per µl in the H1/IC31 and placebo arms, respectively, indicating no difference by study arm (p = 0.85). The median log10 (HIV-1 viral load) slope per day (median −2.9×10^−4^ and −0.54×10^−4^ copies/ml in the H1/IC31 arm and placebo arms, respectively) was also similar by study arm (p = 0.93).

### QuantiFERON status

At baseline, 17/40 (42.5%) of the H1/IC31 and 3/8 (37.5%) of the placebo participants and participants had a positive QFT result. Conversion from QFT negative to QFT positive was observed in 8 of the 23 (34.8%) H1/IC31 recipients, and in none of the placebo recipients. Two of the 17 QFT positive H1/IC31 participants (11.8%) converted to QFT negative by day 182. One initially QFT positive placebo recipient (33.3%) was QFT negative after 182 days. 10 QFT results from 9 H1/IC31 and 1 placebo recipient were missing at day 182 (Table 3).

**Table 3:** QuantiFERON status at baseline and at day 182.

Vaccination group	Study day	n	PositiveN (%)	NegativeN (%)	QuantiFERON conversion	
					Negative toPositive	Positive toNegative
Placebo	Baseline	8	3 (37.5)	5 (62.5)	0	1
	Day 182	7	1 (14.3)	6 (85.7)		
H1/IC31	Baseline	40	17 (42.5)	23 (57.5)	8	2
	Day 182	31	17 (54.8)	14 (45.2)		

### Immunogenicity

**Figure 2:**
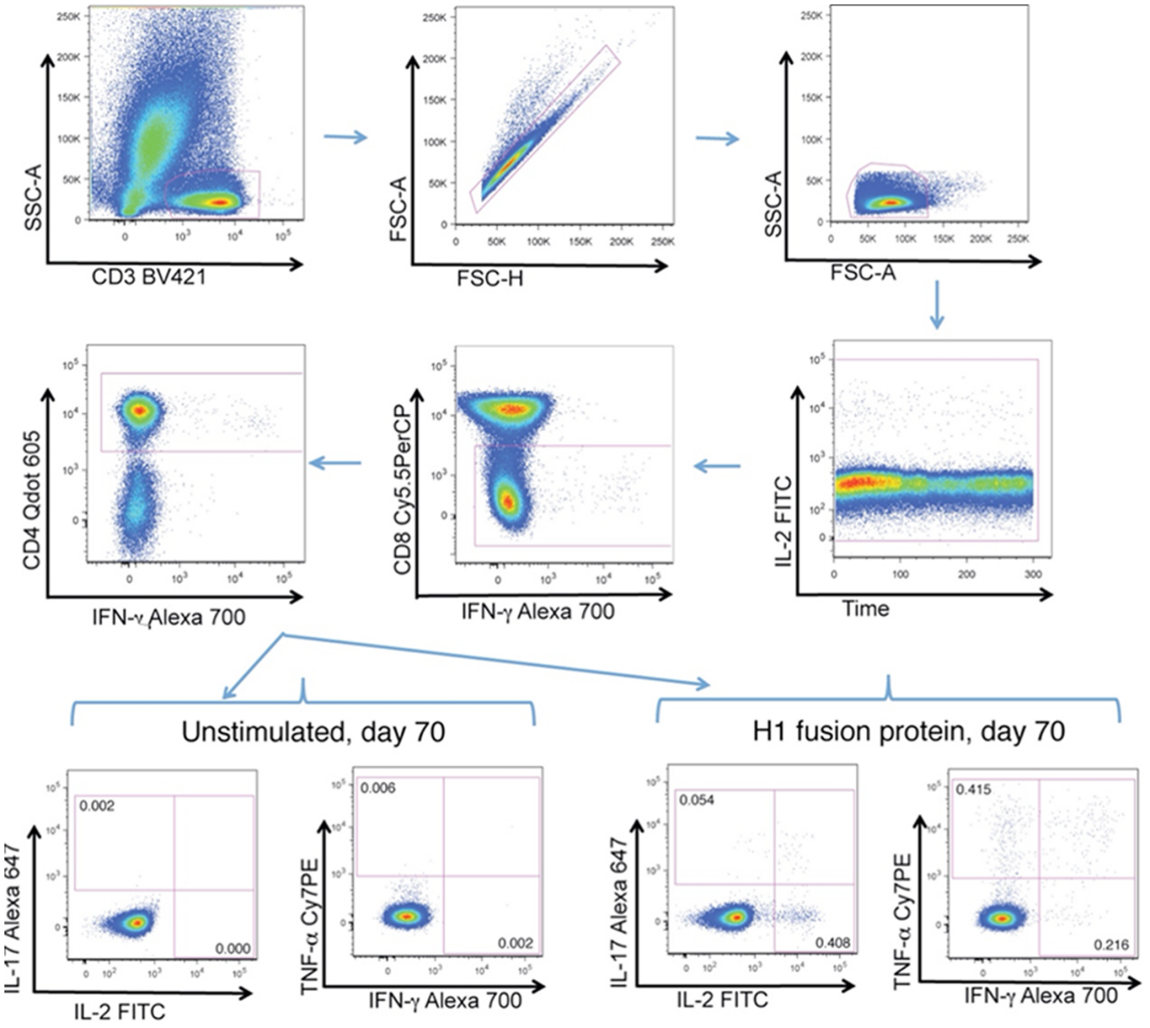
Gating strategy used for flow cytometric analysis of H1-induced T cell cytokine expression. The plots show sequentially the gating hierarchy of one representative sample: CD3+ T cells, single cells, lymphocytes, time, CD8-T cells, which were further gated for CD4+ cells. The plots on the lowest row include the cytokine expression gates for either unstimulated or H1 fusion protein stimulated PBMC at day 70 divided in the co-expression of IL-17 and IL-2 or TNF-α and IFN-γ by CD4+ T cells.

Only samples from the 24 participants from the Bagamoyo Site were used for the immunogenicity analysis. None of the whole blood intracellular cytokine assay specimens from the Tembisa site were useable, as they thawed as a result of the liquid nitrogen tank in which they were stored running dry. The gating strategy for flow cytometry analysis is shown is shown in Fig. 2.

**Figure 3:**
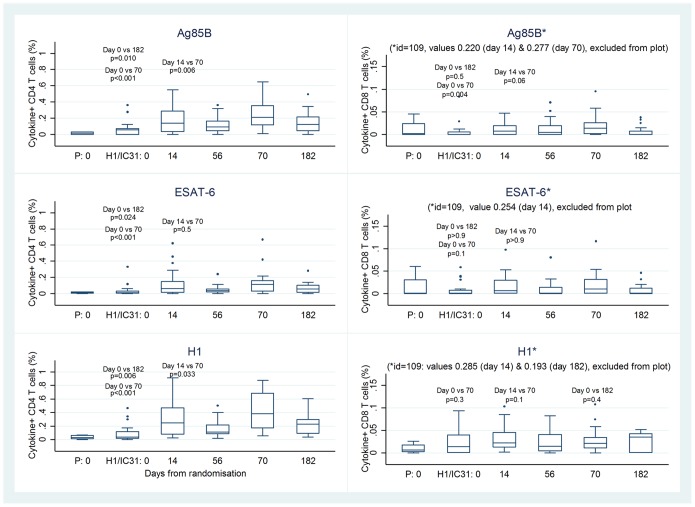
Kinetics of Ag85B, ESAT-6 and H1 specific CD4+ and CD8+ T cells expressing IFN-γ, TNF-α or IL-2 for day 0 for the placebo arm (P: day 0) and over a 6-month period (day 0–182) in the vaccine (H1/IC31) arm detected by a short-term whole blood intracellular cytokine assay.

**Figure 4:**
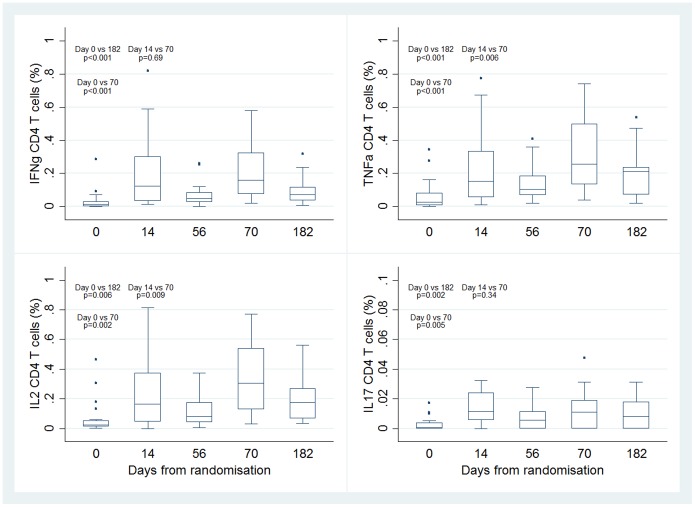
Kinetics of IFN-γ, TNF-α, IL-2 and IL-17 expressing CD4+ T cells after stimulation with H1 over a 6-month period (day 0–182) in the H1/IC31 vaccination group detected by a short-term whole blood intracellular cytokine assay. Footnote: sample size for all measurements at days 0, 14 and 56 was n = 20 and for day 70 and 182 was n = 19.

Among H1/IC31 recipients, the median magnitude of CD4+ T cells expressing one or more cytokines (IFN-γ and/or IL-2 and/or TNF-α) in response to Ag85B, ESAT-6 and H1 were greatest two weeks after the second vaccination (day 70), and remained elevated compared with baseline up to day 182 (Fig. 3). The greatest CD4+ T cell responses were observed after stimulation with H1 (Fig. 3). A similar kinetics of response was seen for CD4+ T cell expressing either IFN-γ, IL-2 or TNF-α in response to H1, and there were no significant IL-17 responses (Fig. 4). Among H1/IC31 recipients the magnitude of CD8+ T cells responses to Ag85B, ESAT-6 and H1 did not exceed pre-vaccination levels two weeks after the second vaccination (Fig. 3).

**Figure 5:**
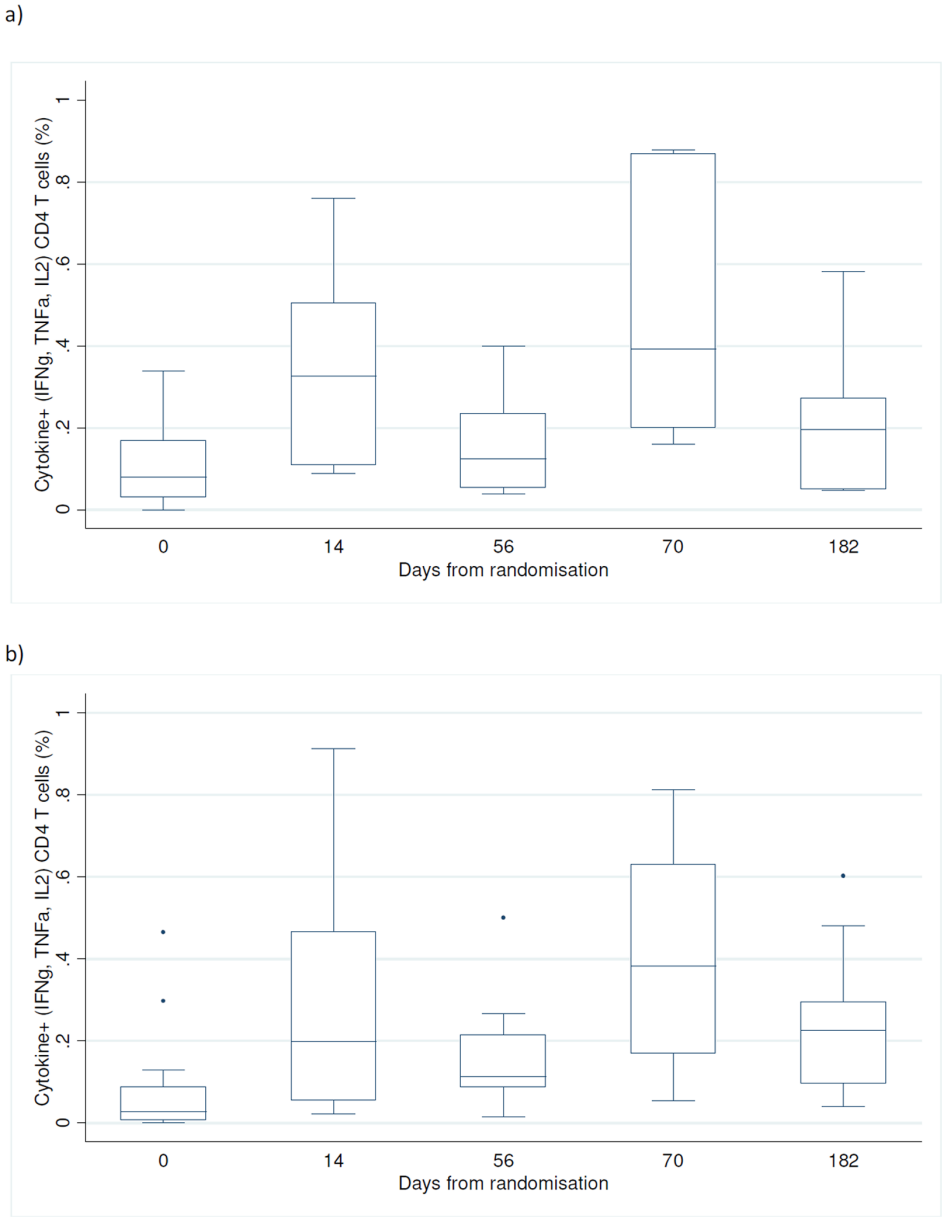
Frequencies of H1 specific CD4+ T cells expressing IFN-γ, TNF-α or IL-2 at day 0, 14, 56, 70 and 182, for vaccination group H1/IC31 group [unstimulated frequencies have been subtracted] for a) QFT positive n = 6 and b) QFT negative n = 14. Footnote: comparison of CD4+ T cell IFN-γ and/or IL-2 and/or TNF-α responses to H1 at day 70 by those QFT positive or negative, p = 0.5.

Among H1/IC31 recipients, the median CD4+ T cell IFN-γ and/or IL-2 and/or TNF-α responses to H1 two weeks post second vaccination (day 70) did not differ by baseline CD4 count (CD4 count<500∶0.34%, ≥500∶0.38%, p = 0.9), or by QFT status (QFT positive: 0.39%, negative 0.38%, p = 0.5, Fig. 5a and 5b).

**Figure 6:**
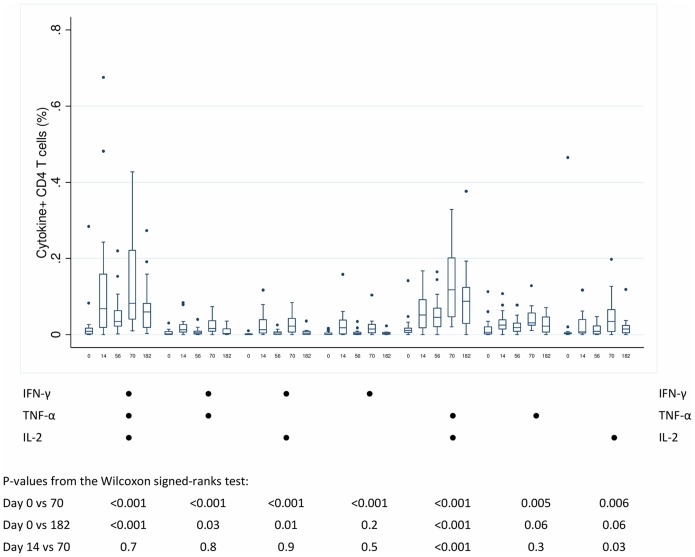
Frequencies of H1 specific CD4+ T cells expressing any combination of IFN-γ, TNF-α and IL-2 at day 0, 14, 56, 70 and 182 in the vaccination group [unstimulated frequencies have been subtracted].

The functional capacity of the CD4+ T cells was assessed in the 7 possible cell subsets producing any combination of the IFN-γ, TNF-α or IL-2 in response to H1 stimulation at day 0, 14, 56, 70 and 182 (Fig. 6). Most reactive CD4+ T cells expressed IFN-γ, TNF-α and IL-2 or TNF-α and IL-2 in response to H1 with a minimum at day 0, a maximum at day 70 and elevated levels at day 182. Single cytokine producing CD4+ T cells most commonly produced IL-2 in response to H1.

### Humoral response determined by Anti-Ag85B-ESAT-6 specific IgG antibody assay

There was no difference in IgG levels by study arms two week post second vaccination (day 70) and 182 (data not shown).

## Discussion

This is the first report on safety and immunogenicity of the H1/IC31 subunit TB vaccine administered to HIV-infected adults with CD4+ lymphocyte counts >350 cells/mm^3^. Key findings are: (i) H1/IC31 was safe and well tolerated; (ii) H1/IC31 did not have a negative impact on CD4+ T cell count or on HIV-1 viral load. (iii) H1/IC31 induced a strong, specific and long lasting, CD4+ T cell response; (iv) H1/IC31 vaccination induced primarily triple (IFN-γ, TNF-α and IL-2) or double (TNF-α and IL-2) positive CD4+ T cells.

There is a concern that in HIV-infected individuals, TB vaccines may promote immune activation leading to increased adverse events and progression of HIV. Vaccination with Modified Vaccinia Ankara virus expressing antigen 85A (MVA85A) [6] [7] [8] and inactivated *M. vaccae* [9] [10] in HIV-infected persons had favourable safety profiles. RUTI vaccination (heat killed *M.tb*, fragmented) of HIV-infected adults was associated with an increased risk of local reactions, including nodules and sterile abscesses [11]. In our study, H1/IC31 vaccination in HIV-infected individuals was tolerable and had a safety profile similar to that seen among H1/IC31 vaccinated, HIV-uninfected, mycobacterially-naïve, BCG vaccinated and latently TB-infected individuals in Europe [4] [5]. In the present study, there was no major change in CD4+ T cell count and HIV-1 viral load trends, similar to reports on vaccination with MVA85A [6] [7] and Aeras 402 [12].

TB vaccines in HIV-infected individuals potentially may be less immunogenic due to HIV-associated immunosuppression. H1/IC31 vaccination of HIV-infected participants with a high CD4 count (>350 cells/mm^3^) induced a specific Th1-type response to the H1 antigen, and its Ag85B and ESAT-6 subunits. We observed some CD4+ T cell IFN-γ responses to the vaccine antigen H1 at enrolment - prior to vaccination. The responses were most prominent in the QFT positive individuals, and probably due to latent TB infections or possibly cross reacting environmental mycobacterial infections. Despite these baseline responses, H1/IC31 vaccination induced significantly higher CD4+ T cell responses that were durable. These results are encouraging and suggest that latent TB infection will not mask an H1/IC31 specific immune response. The magnitude and durability of the Th1 responses in HIV-infected participants, and the lack of humoral response, has similarly been observed in HIV-uninfected participants [4] [5].

In our study, H1/IC31 induced predominately TNF-α/IL-2 and IFN-γ/TNF-α/IL-2 co-expressing CD4+ T cells with a peak two weeks after the second vaccination which remained elevated for up to 6 months. Both cell subsets, in particular the TNF-α/IL-2 double positive subset, have been found to include a high proportion of central memory T cells [13] [14] and to be less differentiated [15], more long lived [15] [16] and functionally superior [17] to IFN-γ single cytokine expressing T cells. The protective role of polyfunctional CD4+ T cells against tuberculosis infection is still debated. However, several studies have observed a positive correlation between protection against disease and induced T cell polyfunctionality, particularly those expressing IL-2, which is associated with long-term persistence of primed CD4+ T cells *in vivo* [14] [18] [19] [20] [21]. The TNF-α/IL-2 central memory T cell population may be of particular relevance for the activity of these vaccines. In a recent murine study TNF-α/IL-2 central memory T cells were implicated in the long-term control of chronic TB infection [22]. This predominance of the TNF-α/IL-2 subset seems to be characteristic for the IC31 and CAF01 adjuvant. In comparison, the MVA85A, M72/AS01 and M72/AS02 vaccines primarily induced polyfunctional IFN-γ/TNF-α/IL-2 CD4+ T cells [23] [24], while Aeras 402 induced both polyfunctional CD4+ and CD8+ T cells [25].

The role of Th17 cells producing IL-17 is the subject of ongoing debate. Reports suggest that IL-17 producing T cells support a balanced immune response improving the protective efficacy of novel vaccines [24] [26]. In our study, H1/IC31 induced IL-17 mono- or co-expressing CD4+ T cells that were barely detectable post vaccination and contrasts with MVA85A which induced IL-17 producing T cells [24].

H1/IC31 vaccine induced poor CD8+ T cell responses, which has likewise been described for other vaccine candidates [6] [23]. These findings may be explained by methodological issues of the *ex vivo* assay system, such as the possible suboptimal length of the peptides used in the assay (15 amino acids) or an inadequate limit of detection [6] [23].

As formerly reported, H1/IC31 vaccination can lead to conversion of the QFT result because of its ESAT-6 component [4] [5]. In our study, this phenomenon occurred in one third of initially QFT negative participants. However, reversions suggest that some of the observation might also be attributed to within-subject variability of the QFT assay [27]. The concern that H1/IC31 can interfere with IFN-γ release assays (IGRA) such as QFT for detection of LTBI has becomes less important since the WHO does not recommend IGRA to replace tuberculin skin tests in resource-constrained settings [28], where the vaccine would be broadly used. Furthermore, new tests using diagnostic markers other than ESAT-6 for disease screening are under development and have the potential to eliminate the issue of interference with the vaccine for low- and high-burden countries [29].

New subunit TB vaccines should boost the efficacy of BCG and prevent TB infection and prevent latent TB infection from progressing to active disease. Both mechanisms are essential for the control of the TB epidemic. In order to achieve a better post-exposure protection, the Statens Serum Institut has added a latency-associated antigen (Rv2660c) to the H1/IC31 backbone, to create a multistage vaccine, H56 (Ag85B-ESAT-6-Rv2660c) [30]. Our trial of H1/IC31 provides valuable information to support the clinical development of the novel multistage vaccine H56. Particularly important is that H1/IC31 induces multifunctional T cells expressing TNF-α and IL-2, markers of central memory T cells, which are required for long-term protection against both reactivation and infection [22]. This is critical for the vaccination of vulnerable populations such as HIV-infected individuals who often have a high level of exposure to TB and a relatively high prevalence of latent TB infection. Inevitably, the multistage H56 vaccine should also be evaluated in HIV-infected individuals to ensure that it confers durable immune protection in this target group.

The present study had a number of limitations. The study was not powered to show difference between subgroups, particularly by baseline QFT status. The whole blood intracellular cytokine staining data from one site and IFN-γ EliSpot assay data from both sites did not meet the internal quality control standards, which limits comparability to other TB vaccine trials. The restriction to HIV-infected individuals with CD4+ lymphocyte counts greater than 350 cells/mm^3^ was necessary because of safety concerns, but limits the generalizability of the results.

In conclusion, the intramuscular injection of the adjuvanted TB subunit vaccine H1/IC31 was safe and immunogenic in HIV-infected adults, with CD4+ lymphocyte counts greater than 350 cells/mm^3^, from TB endemic areas.

## Supporting Information

S1 Protocol **Study protocol of the study ‘Phase II Double-Blind, Randomized, Placebo-Controlled Study to Evaluate the Safety and Immunogenicity of H1/IC31, an adjuvanted TB Subunit Vaccine, in HIV-Infected Adults with CD4+ Lymphocyte Counts Greater than 350 cells/mm^3^.’**
(PDF)

S1 Checklist **Consolidated Standards of Reporting Trials (CONSORT) checklist.**
(DOC)
