# Supplementary material for: Safety and Immunogenicity of H1/IC31®, an Adjuvanted TB Subunit Vaccine, in HIV-Infected Adults with CD4+ Lymphocyte Counts Greater than 350 cells/mm3: A Phase II, Multi-Centre, Double-Blind, Randomized, Placebo-Controlled Trial
Source: PLoS One. 2014 Dec 9;9(12):e114602. doi: 10.1371/journal.pone.0114602 (PMC4260867; doi:10.1371/journal.pone.0114602)
Supplement: S1 Protocol — Study protocol of the study ‘Phase II Double-Blind, Randomized, Placebo-Controlled Study to Evaluate the Safety and Immunogenicity of H1/IC31, an adjuvanted TB Subunit Vaccine, in HIV-Infected Adults with CD4+ Lymphocyte Counts Greater than 350 cells/mm3.’ (PDF) [file pone.0114602.s001.pdf]

CLINICAL PROTOCOL

PHASE II DOUBLE-BLIND, RANDOMIZED, PLACEBO-CONTROLLED STUDY TO EVALUATE THE SAFETY AND IMMUNOGENICITY OF H1/IC31®, AN ADJUVANTED TB SUBUNIT VACCINE, IN HIV-INFECTED ADULTS WITH CD4+ LYMPHOCYTE COUNTS GREATER THAN 350 CELLS/MM^3

Trial code: Aurum 102 / THYB-05

VERSION 2.1

15 February 2011

Confidentiality

This document contains confidential information. The contents may not be used, divulged or published without prior written consent of Statens Serum Institut and The Aurum Institute.
This information cannot be used for any other purpose than the conduct and evaluation of the clinical investigation by the investigator(s), the regulatory authorities and members of the ethics committees.

Protocol Synopsis

**Title**	Phase II Double-Blind, Randomized, Placebo-Controlled Study to Evaluate the Safety and Immunogenicity of H1/IC31®, an adjuvanted TB Subunit Vaccine, in HIV-Infected Adults with CD4+ Lymphocyte Counts Greater than 350 cells/mm^3
**Trial code**	Aurum 102 \ THYB-05
**Trial phase**	Phase II
**Primary objective**	To evaluate the H1/IC31® TB vaccine in HIV-infected adults for:<br>• Safety<br>• Induction of cellular and humoral immunity
**Secondary objective**	To describe the effect of the H1/IC31® TB vaccine in HIV-infected adults on:<br>• CD4+ lymphocyte counts<br>• HIV viral loads
**Exploratory objective**	To evaluate innate and adaptive immune response to H1/IC31® TB vaccine in HIV-infected adults using transcriptomics.
**Primary variables**	The primary variables will be based on medical examinations, blood and urine samples.<br><br>**Safety Variables**<br>The primary variables will be based on medical examinations, blood and urine samples.<br><br>**Physical examination**<br>• Local adverse events, including pain, erythema, induration, swelling, itching, nodules, and local functional limitations<br>• Systemic adverse events, including diffuse erythema, urticaria, asthma, angioedema, fatigue, fever, arthralgia, myalgia, hoarseness, dizziness, malaise, sweating, nausea, arrhythmia, headache, and anaphylaxis<br>• General examination including ENT, cardiovascular, pulmonary, neurological, gastrointestinal, urogenital, and dermatological systems<br><br>**Laboratory safety tests**<br>• Blood samples will be tested for RBC, differential WBC, platelets, haemoglobin, hematocrit, ASAT, ALAT, alkaline phosphatase, lactate dehydrogenase, γGT, albumin, bilirubin, creatinine, glucose, CD4 count, viral load.<br>• Urine samples will be tested for glucose, protein and sediment.<br><br>**Immunogenicity assessments**<br>• Detection by ELISPOT of IFNγ spot-forming cells in stimulated PBMCs.<br>• Detection by ELISA of IFNγ, TNF-α and IFNγ production in supernatants of stimulated PBMCs<br>Detection by ELISA of IgG antibodies to H1 in serum or plasma.
**Secondary variables**	CD4+ T-lymphocyte cell counts measured by flow cytometry<br>HIV-1 viral loads measured by Roche amplicor
**Exploratory objective**	• Analysis of changes in transcriptional gene expression pattern in whole blood before and after vaccination measured by the SOLiD system<br>• Characterization of H1/IC31 vaccine antigen specific cellular immunity in terms of chemokines/cytokine mRNA expression using quantitative real time PCR<br>• Analysis of cytokines produced by peripheral blood mononuclear cells after H1/IC31 vaccine component stimulation using multiplex cytokine/chemokine luminex assays<br>• Profiling of intracellular cytokine production by peripheral blood mononuclear cells after H1/IC31 vaccine components stimulation<br>Characterisation of H1/IC31 vaccine specific central memory responses by proliferation assays and multi-colour flow cytometry
**Trial design**	This is a Phase II, randomized, double-blind, placebo-controlled trial evaluating the safety and immunogenicity of H1/IC31 administered to HIV-infected adult subjects with no evidence of TB disease.

	Randomization will be performed on Study Day 0 prior to the administration of the study vaccine. Subjects will be randomly allocated in a 5:1 ratio to receive a total of two doses of either H1/IC31 or placebo vaccination, given at Study Day 0 and at Day 56.
**Inclusion criteria**	1. Age between 18 and 55 years<br>2. Has laboratory evidence of HIV infection, defined as a positive HIV-1 ELISA test plus a positive confirmatory test (e.g., a second HIV-1 ELISA, PCR, or rapid ELISA)<br>3. Have two CD4+ lymphocyte counts, at least four days apart within the screening period, greater than 350 cells / mm^3.<br>4. Not currently receiving antiretroviral drugs.<br>5. Healthy based on medical examination/history at the inclusion<br>6. Females: Ability to avoid pregnancy during the trial: Women physically capable of pregnancy (not sterilized and still menstruating or within 1 year of the last menses if menopausal) must avoid pregnancy from 21 days prior to administration of the study vaccine through the end of the study. Acceptable methods of avoiding pregnancy include a sterile sexual partner, sexual abstinence (not engaging in sexual intercourse), or use of a combination of at least two forms of acceptable contraception: hormonal contraceptives (oral, injection, transdermal patch, or implant), vaginal ring, intrauterine device (IUD), and the use of a condom or a diaphragm; or the use of a condom or a diaphragm combined with spermicide.<br>7. Is able to carry out activities of daily living independently<br>8. Is able and willing to comply with study procedures and to complete follow-up period as required by the protocol<br>9. Is able and willing to commit to avoid elective surgery for the duration of the study<br>10. Is prepared to grant authorized persons access to the medical records<br>11. Has signed informed consent
**Exclusion criteria**	1. Acute illness<br>2. Fever ≥37.5°C at enrolment<br>3. Evidence of or suspected active TB<br>4. Used immunosuppressive medication within 42 days prior to randomization (inhaled and topical corticosteroids are permitted.)<br>5. Received immunoglobulin or blood products within 42 days prior to randomization<br>6. Medical history that in the opinion of the investigator may compromise the evaluation of safety of the subject in the study (e.g., diabetes, seizure disorder, sickle cell disease)<br>7. Pregnant or breastfeeding female, or intending to become pregnant during the study period<br>8. Received any investigational drug therapy or vaccine within 182 days prior to randomization<br>9. Known hypersensitivity to any of the vaccine components<br>10. Laboratory parameters outside of normal range considered clinically relevant
**Trial population**	The study will be conducted at clinical trial sites in Bagamoyo District, Tanzania and Ekurhuleni district Johannesburg South, Africa. One study group is planned, with an anticipated enrolment of 48 subjects split evenly between the two sites.
**Number of subjects**	48
**Country**	South Africa and Tanzania
**Investigational product**	H1/IC31®
**Dosage**	H1 antigen: 50 µg<br>IC31 adjuvant: 500 nmol KLK and 20 nmol ODN1a
**Dosage volume**	0.5 ml
**Administration**	Two intramuscular injections, one on day 0 and one on day 56

List of abbreviations

γGT	Gamma glutamyl-transpeptidase
AE	Adverse event(s)
Ag85B	Antigen 85B
AIDS	Acquired Immune Deficiency Syndrome
ALAT	Alanine aminotransferase
AM	Abbreviated medical examination
Aurum	The Aurum Institute
BCG	Bacillus Calmette-Guérin
CBC	Complete blood count
CFSE	Carboxyfluorescein diacetate succinimidyl ester
CPK	Creatine phosphokinase
CPT	Cell Preparation Tube
CRF	Case Report Form(s)
CTA	Clinical Trials Application
DAIDS	National Institute of Health Division of AIDS
DSMB	Data Safety Monitoring Board
EC	Ethics Committee
ELISA	Enzyme Linked Immunosorbent Assay
ELISPOT	Enzyme Linked Immunosorbent Spot Assay
ESAT-6	Early Secretory Antigenic Target
FDA	United States Food and Drug Administration
FM	Full medical examination
FU	Follow up
GCP	Good clinical practice(s)
H1	Hybrid 1 (Ag85B-ESAT-6)
H1/IC31®	Ag85B-ESAT-6 + IC31
HBV	Hepatitis B virus
HCV	Hepatitis C virus
HIV	Human immunodeficiency virus
IB	Investigator’s Brochure
IC31	KLK, ODN1a
ICH	International Conference on Harmonisation
ICS	Intracellular Cytokine Staining
IFNγ	Interferon-gamma
IgG	Immunoglobulin G
IgM	Immunoglobulin M
IM	Intramuscular
IRB	Institutional Review Board
KLK	KLKLLLLLKLK
LUMC	Leiden University Medical Center
MCC	Medicines Control Council (South Africa)
mL	Milliliter(s)
Mtb	*Mycobacterium tuberculosis*

ODN1a	Oligodeoxynucleotide
PBMC	Peripheral blood mononuclear cell(s)
PCR	Polymerase chain reaction
PHA	phytohemagglutinin
PPD	Purified protein derivative
pys	patient years
QFT-GIT	QuantiFERON®-TB Gold In Tube, Cellestis Ltd.
RBC	Red Blood Cells
SAE	Serious adverse event(s)
SSI	Statens Serum Institut
SST	Serum separator tube
SUSAR	Suspected unexpected serious adverse reaction
TB	Tuberculosis or tuberculous
TNF	Tumor necrosis factor
TST	Tuberculin skin test
WBC	White blood cell

Protocol Signature Page

By signing this document, I declare that I have read and understand the protocol specified above and agree on its content. I agree to ensure this study is conducted according to the protocol, associated study documents, ICH GCP guidelines, the Declaration of Helsinki, and pertinent individual country laws and regulations. I shall not disclose the information contained in this protocol or any results obtained from this study without written authorization from a duly authorised person at The Aurum Institute and Staten Serum Institut.

Protocol Chair	Prof. Gavin Churchyard	
		------------------------------------<br>*Date and signature*
Protocol Co-Chair	Prof. Mark Doherty	
		------------------------------------<br>*Date and signature*
Clinical Trial Manager	Dr. Peter Bang	
		------------------------------------<br>*Date and signature*
Sponsor Representative	Dr. Ingrid Kromann	
		------------------------------------<br>*Date and signature*
Medical Advisor	Dr. Trine R. Nielsen	
		------------------------------------<br>*Date and signature*
Principal Investigator	Dr. Lesego Khantsi	
		------------------------------------<br>*Date and signature*
Sub-Investigator	Dr Chanel Maree	
		------------------------------------<br>*Date and signature*
Principal Investigator	Dr. Klaus Reither	
		------------------------------------<br>*Date and signature*
Sub-Investigator	Dr. Humphrey Shao	
		------------------------------------<br>*Date and signature*

Addresses

**Sponsor**		
Protocol Co-chair	Mark Doherty<br><br>Statens Serum Institut<br>Artillerivej 5<br>DK-2300 Copenhagen S	Phone: +45 3268 3844<br>Fax: +45 3268 3035<br>e-mail: tmd@ssi.dk
Sponsor Contact	Peter Bang<br><br>Statens Serum Institut<br>Artillerivej 5<br>DK-2300 Copenhagen S	Phone: +45 3268 8191<br>Fax: +45 3268 3972<br>e-mail: pba@ssi.dk
Sponsor Representative	Ingrid Kromann<br><br>Statens Serum Institut<br>Artillerivej 5<br>DK-2300 Copenhagen S<br>Denmark	Phone: +45 3268 8252<br>Fax: +45 3268 3972<br>e-mail: ikr@ssi.dk
Medical Adviser	Trine R. Nielsen<br><br>Statens Serum Institut<br>Artillerivej 5<br>DK-2300 Copenhagen S<br>Denmark	Phone: +45 3268 3436<br>Fax: +45 3268 3973<br>e-mail: trn@ssi.dk
**The Aurum Institute**		
Protocol Chair	Gavin Churchyard<br><br>The Aurum Institute<br>29 Queens Road<br>Parktown<br>South Africa	Phone: +27 10 590 1306<br>Fax: + 27 86 613 3718<br>e-mail: GChurchyard@auruminstitute.org
Clinical Trial Manager	Lynn Katsoulis<br><br>The Aurum Institute<br>29 Queens Road<br>Parktown<br>South Africa	Phone: +27 10 590 1392<br>Fax: + 27 86 613 3718<br>e-mail: LKatsoulis@auruminstitute.org
Principal Investigator	Lesego Khantsi<br><br>The Aurum Institute<br>Tembisa Hospital<br>Flint Mazibuko Street<br>Tembisa<br>South-Africa	Phone: +27 11 926 8445<br>Fax: +27 11 926 2139<br>e-mail: lkhantsi@auruminstitute.org

**Ifakara Health Institute**		
Principal Investigator	Klaus Reither<br><br>Ifakara Health Institute / Bagamoyo Research and Training Centre<br>Bagamoyo<br>Tanzania	Phone: +255 232 440 293<br>Fax: +255 232 440 064<br>e-mail: Klaus.Reither@unibas.ch
**Study Monitoring**		
Swiss Tropical and Public Health Institute	Christian Burri<br><br>Medicines Research<br>Eulerstrasse 54<br>Basel<br>4051<br>Switzerland	Phone: +41 61 225 2661<br>Fax: +41 61 225 2678<br>e-mail: Christian.Burri@unibas.ch
**Safety Reporting**		
	Statens Serum Institut<br>Artillerivej 5<br>DK-2300 Copenhagen S	Fax: + 45 3268 3973<br>Mobile: + 45 2567 0914<br>e-mail: clin.trial@ssi.dk

Emergency Telephone Numbers

Protocol Chair	Gavin Churchyard	Office: +27 10 590 1306<br>Mobile: +27 82 338 3557
Trial Manager	Lynn Katsoulis	Office: +27 10 590 1392<br>Mobile: +27 83 393 4232
Sponsor Contact	Peter Bang	Office: +45 3268 8191<br>Mobile: +45 2096 9190
Study Monitor	Christian Burri	Office: +41 61 225 2661<br>Mobile: +41 79 742 4681
Medical Advisor	Trine R. Nielsen	Office: +45 3268 3436
Serious Adverse Event Notification:		Fax: + 45 3268 3973<br>Mobile: +45-2567 0914<br>e-mail: clin.trial@ssi.dk

Table of Contents

Page

Protocol Synopsis ... 2
List of abbreviations ... 4
Protocol Signature Page ... 6
Addresses ... 7
Emergency Telephone Numbers ... 9
Table of Contents ... 10
1. Introduction ... 13
1.1 Description of H1/ IC31® sub-unit vaccine ... 14
1.2 Non- clinical experience with IC31® and H1/IC31® sub-unit vaccine ... 14
1.3 Clinical experience with H1/IC31® sub-unit vaccine ... 15
2. Trial objectives ... 17
2.1 Primary objectives ... 17
2.2 Secondary objectives ... 17
2.3 Exploratory objectives ... 17
2.4 Primary variables ... 17
2.4.1 Laboratory safety tests: ... 17
2.4.2 Immunogenicity variables: ... 18
2.5 Secondary variables ... 18
2.6 Exploratory variables ... 18
3. Investigational plan ... 19
3.1 Overall design ... 19
3.2 Study population ... 19
3.3 Recruitment and Informed consent ... 19
3.4 Inclusion criteria ... 20
3.5 Exclusion criteria ... 20
3.6 Table 2. Schedule of events ... 21
3.7 Details of visits ... 22
3.8 Handling of blood samples ... 25
3.8.1 Sample collection ... 25
3.8.2 PBMC isolation and storage ... 25
3.9 Laboratory immunogenicity analyses ... 25
3.9.1 ELISpot and ELISA ... 26
3.9.2 Quantitative real time PCR analysis of PBMC after stimulation ... 26
3.9.3 Multi-plex quantification of cytokine and chemokines in PBMC after stimulation ... 26
3.9.4 Analysis of intracellular cytokines by multi-colour flow cytometry after stimulation ... 26
3.9.5 Proliferation assay of effector central memory cellular immunity after stimulation ... 27
3.9.6 Whole blood intracellular cytokine staining assay ... 27
3.9.7 Ex-vivo Ki67 assay ... 27
3.9.8 IgG assay ... 28
3.9.9 Transcriptomics ... 28
3.10 Time Schedule ... 28

3.11 Withdrawal and predetermined reasons for discontinuation 28
3.12 Pregnancy 28

4. Investigational product 29
4.1 Treatments administered 29
4.2 Packing and labelling 30
4.3 Doses and administration 30
4.4 Vaccine storage and dose preparation 30
4.5 Transport of trial products 31
4.6 Treatment Group Assignment or Randomization 31
4.7 Replacement of withdrawn subjects 31
4.8 Study Blind 31
4.9 Unblinding for Clinical Emergencies 31
4.10 Treatment compliance, drug accountability 32
4.11 Precautions 32
4.12 Concomitant medication 32
4.13 Prohibited medication 32

5. Adverse events 33
5.1 Definitions and terms 33
5.2 Adverse event 33
5.3 Adverse reaction or suspected adverse reaction 33
5.4 Unexpected Adverse Drug Reaction 33
5.5 Seriousness criteria 33
5.6 Unexpected adverse reaction 34
5.7 Documentation of adverse events 34
5.7.1 Causality 34
5.7.2 Severity 34
5.7.3 Outcome 35
5.7.4 Seriousness 35
5.8 Reporting of adverse events 35
5.8.1 Reporting SAEs to SSI 35
5.8.2 SSI's expedited reporting of SUSARs to the regulatory authorities and ethics committees 35
5.8.3 Safety reporting to the relevant ethics committees 37

6. Stopping Rules 37

7. Data management and statistical analysis 38
7.1 Data management 38
7.2 Statistical analysis 38
7.3 Primary endpoints 38
7.3.1 Adverse events: 38
7.3.2 Laboratory safety tests: 38
7.3.3 Immunogenicity variables: 39
7.4 Secondary endpoints 39
7.5 Exploratory endpoints 39
7.6 Sample Size 39
7.6.1 Sample size for the primary endpoints 39
7.6.2 Sample size for immunogenicity endpoints 41

8. Good Clinical Practice considerations 42
8.1 Applicable guidelines 42
8.2 Subject information and informed consent 42

8.3 Regulatory and ethics committee approval 42
8.4 Subject data protection 42
8.5 Amount of blood drawn 42
8.6 Compensation of volunteers and investigational institution 42
8.7 Investigators responsibilities 43
8.8 Indemnity statement: 43
8.9 Training 43
8.10 Monitoring 43
8.11 Audit and Inspection 43
8.12 Definition of source data 43
8.13 Archiving 44
8.14 Agreement and financial settlement 44

9. Confidentiality and disclosure 44

10. References 45

11. Appendices 46

1. Introduction

Despite more than a half-century of widespread vaccination, tuberculosis (TB) remains one of the world's most serious infectious diseases, claiming nearly 2 million lives every year [(1)]. TB is typically passed from an infected person to a new host by inhalation of bacteria in sputum exhaled by an infected person. The characteristic symptom of TB is a prolonged cough, which is induced by tissue destruction in the lung by the pathogen. Unfortunately, cough and fever are relatively non-specific symptoms, and the period of time between infection and appearance of symptoms can be anywhere from a few months to several decades. In addition to improved public health control measures, such as intensified case finding, an effective TB vaccination programme is required to meet the Millennium Development Goals and Stop TB partnership's target for TB elimination by 2050.

The majority of TB is now restricted to the world's poorest countries, and ongoing studies suggest that in these countries as many as 30-40% of adolescents are already latently infected and thus at risk of disease. In addition to TB-related mortality, the resources used to combat TB and the productive years lost to TB morbidity inflict a punishing toll on the economies of TB-endemic countries, helping mire them in poverty. The last factor worsening the epidemic is the observation that HIV positivity is a significant risk factor for the development of TB and the prognosis for those with HIV/TB co-infection is particularly poor.

BCG (*Mycobacterium bovis Calmette Guerin*) is an attenuated strain of *Mycobacterium bovis* derived through more than 13 years of continuous *in vitro* passage. It remains the only approved vaccine against TB and more than 3 billion people have received BCG to date. However, while studies have repeatedly demonstrated that vaccination with BCG in infancy is highly effective against TB in children, the major burden of TB is associated with pulmonary disease in later life, against which the vaccine has proven less effective. There is thus a huge need for a new, effective boost or therapeutic TB vaccine in adults that can be made available to low income settings. In addition, it needs to be safe in those who are HIV-infected. BCG fails both of these requirements, and so a new vaccine is the only way forward [(2)].

Testing new TB vaccines in Africa is thus of the highest priority. Several new TB vaccines are being developed, including recombinant BCG vaccines, attenuated *Mycobacterium tuberculosis* vaccines, DNA-based vaccines and subunit vaccines [(3)]. Living mycobacterial vaccines pose significant health risks in HIV-infected individuals, and although this can be addressed by auxotrophic mutants, significant regulatory barriers imposed on genetically modified organisms have impeded efficient development of living mycobacterial vaccines. DNA vaccines have so far been disappointing with regard to immunogenicity and protection in model studies [(3)]. Not surprisingly, then, three of the most advanced vaccines are all subunit vaccines, and two of these comprise recombinant proteins.

The subunit vaccine approach builds on the concept of stimulating the immune response to a number of selected antigens delivered in the form of recombinant antigens. The Statens Serum Institut has cloned and screened 250 antigens from *Mycobacterium tuberculosis* and selected a small number of the most antigenic candidates. Among these are the early secretory antigenic target (ESAT-6) and antigen 85 (Ag85B). Both are strongly recognized T-cell antigens in the first phase of infection, that have demonstrated protective efficacy in animal models, and both contain numerous well recognized epitopes, recognized in TB patients [(4, 5)].

As compared to conventional vaccines, the new recombinant protein are poorly immunogenic when administered alone, but when co-administered with an adjuvant have been shown to elicit strong immune responses.

Aluminium salts are by far the most widely used adjuvants in vaccines to date despite their mechanism of action not yet being fully understood. It is known, however, that they do not optimally support the development of Th1 cells, which are thought to be required for protective immunity against tuberculosis. This has prompted the development of new adjuvants [(6)].

Intercell AG has developed a new adjuvant called IC31®, a two-component adjuvant composed of a cationic polyaminoacid, KLK, and the oligodeoxynucleotide ODN1a in a specific molar ratio of 25:1 KLK to ODN1a. The combination KLK/ODN1a has been shown to induce potent cellular and humoral responses using different peptides and antigens [(7)].

The vaccine we propose to test (H1/IC31®) has already been shown to be safe and immunogenic in humans including those previously infected with TB [(8)] and can be produced in bulk very cheaply. A planned study in Ethiopia (THYB-04) will determine optimal dosing in an African TB-endemic population. This study (Aurum102/THYB-05) will examine the already confirmed dosing regimen in HIV-infected individuals, to determine safety and immunogenicity. H1/IC31® can be lyophilized, potentially leading to a cheap, bulk product that can be delivered throughout the developing world, including anywhere in Africa. If such a vaccine were effective, the public health benefit would be enormous.

1.1 Description of H1/ IC31® sub-unit vaccine

Ag85B-ESAT6 is a recombinant fusion protein of Ag85B and ESAT-6, which are both secreted, immunodominant proteins of *Mycobacterium tuberculosis.* IC31® is a two-component adjuvant system developed by Intercell AG, composed of the cationic polyaminoacid KLK and the oligodeoxynucleotide ODN1a in specific molar ratios of 25:1 KLK to ODN1a. KLK is a cationic peptide composed of the amino acids Lysine (K) and Leucine (L). ODN1a is a single-strand oligodeoxynucleotide based on alternating sequences of the nucleic acids inosine and cytidine. The IC31® adjuvant, composed of KLK and ODN1a as well as the final vaccine are manufactured by the Statens Serum Institut (SSI).

1.2 Non- clinical experience with IC31® and H1/IC31® sub-unit vaccine

The nonclinical pharmacology and immunology studies were performed at SSI (Denmark), at the Biomedical Primate Research Center (BPRC; Holland), and at the University of Geneva (Switzerland). The pharmacology studies (non-GLP) were performed in mice (including neonate mice), guinea pigs and monkeys (cynomolgus and rhesus). Good Laboratory Practice (GLP) compliant toxicology studies were performed in three accredited facilities: Scantox (Denmark), the Laboratory of Pharmacology and Toxicology (Germany) and the BPRC. The single-dose toxicity studies were performed in mice and rabbits, repeated dose toxicity studies in mice, rabbits and monkeys, and mutagenicity studies were performed in *Salmonella typhimurium.*

Additional information on preclinical non-GLP studies on the ESAT-6/Ag85B subunit vaccine is detailed in the investigator’s brochure. Briefly, the activity of antigen adjuvanted with IC31® after vaccination has been investigated in different animal species/strains with different genders, ages, dosages and routes of administration (intramuscular administration, which is the administration intended for human use, and subcutaneous administration). No systemic toxicity was observed. Only local, transient, test article related changes were observed [(9)].

The induction of an immune response after immunisation was demonstrated by release of IFN-□ in cell cultures after restimulation with ESAT-6/Ag85B, generation of IgG antibodies against ESAT-6/Ag85B in mice, and a positive skin-test response in immunised guinea pigs. A protective immune response was obtained after challenge with *M. tuberculosis* in mice measured as reduced bacterial load in the lungs and spleen, and in guinea pigs as increased median survival time.

It was assessed that the existing preclinical toxicology, immunogenicity and safety data was adequate support for the initiation of clinical trials in humans**.**

1.3 Clinical experience with H1/IC31® sub-unit vaccine

In the first phase 1a trial, THYB-01 at Leiden University Medical Center (LUMC), 36 mycobacterially-naïve male volunteers received at day 0 and 2 months the following dosage:

group 1: 50 µg H1
group 2: 50 µg H1 + 100 nmol KLK + 4 nmol ODN1a
group 3: 50 µg H1 + 500 nmol KLK + 20 nmol ODN1a

Main results of this trial [8] were as follows: No serious adverse events occurred, and although some systemic adverse events were reported, most were considered to be unrelated to the administration of the vaccine. Two mild, adverse events that disappeared within one day were reported where relatedness to the vaccine could not be excluded. The only significant vaccine-related response observed in some subjects was pain and bruising at the site of the injection one day after administration of the vaccine. In all cases, the local soreness was mild (grade I), disappeared within a day, did not hinder function of the extremity, did not require analgesics, and was not accompanied by local induration, lymphadenitis, a febrile reaction or systemic symptoms.

To further evaluate the safety of vaccination, extensive haematological and biochemical assessments were conducted before starting, at the time of each of the two vaccinations and after completing long-term follow-up. The vaccine did not noticeably affect any haematological or biochemical measurement.

The vaccination induced specific, strong and persistent Th1-type immune reactivity to the vaccine antigen. Moreover, a single dose of antigen adjuvanted with IC31® induced a significant cellular immune response that was further amplified by booster vaccination (Figure 1). In general, T-cell responses were most prominent against the H1 hybrid protein and secondarily against the Ag85B component and, following the booster administration to those receiving the high dose adjuvant vaccine, were sustained during a 131-week follow-up suggesting induction of potent immunological memory. With respect to humoral responses induced by vaccination, little or no response was detectable against different vaccine components.

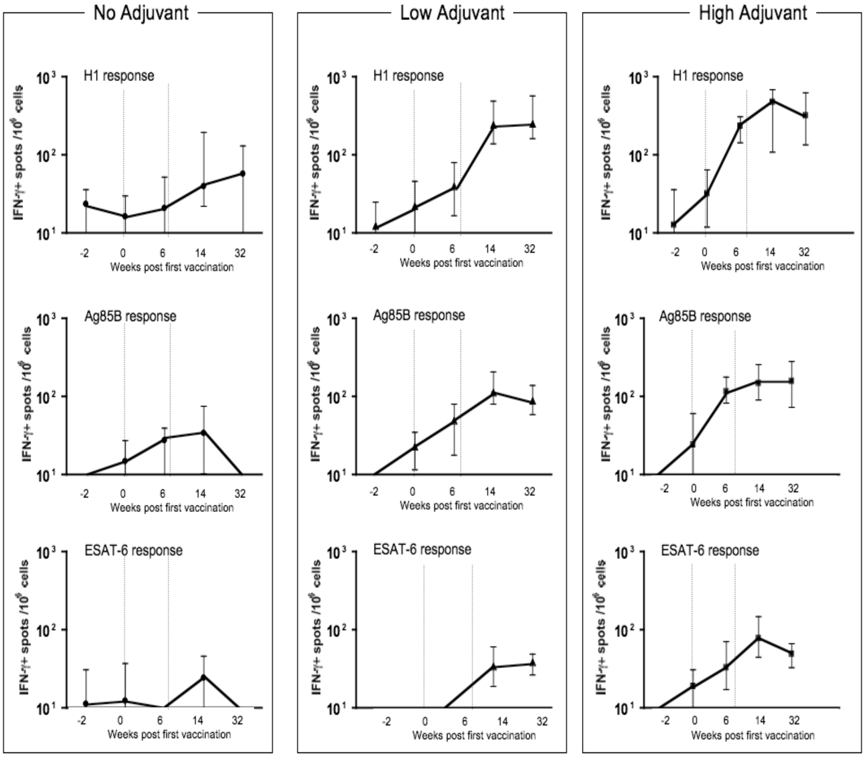

Figure 1. Results from THYB-01: median IFN-□□production in response to vaccine antigen and vaccine components over time. The dotted vertical lines indicate time of vaccinations [(8)].

Based on the above data, the next trial, designated THYB-02, was initiated in 2008 also at LUMC. In this study 10 BCG vaccinated volunteers and 10 prior or latently TB infected individuals received 50 µg H1 + 500 nmol KLK + 20 nmol ODN1a at time 0 and 2 months. The main conclusions are as follows: No serious adverse events were reported but some probably vaccine-related local adverse events were reported, which included injection site movement impairment, injection site pain, injection site nodule and injection site swelling. A few probably vaccine-related systemic adverse events were reported, which included fatigue, malaise and feeling cold. These adverse events were minor and rapidly resolved without intervention.

Immune responses, as assessed by ELISpot and ELISA showed significant increases in IFN-□ induced by H1, ESAT-6 and Ag85B in the BCG group while both H1 and Ag85B yielded significant increases in IFN-□□in the Infection group [(10)].

A third trial, designated THYB-03, also using 50 µg H1 + 500 nmol KLK + 20 nmol ODN1a administered at time 0 and 2 months was initiated at the Armauer Hansen Research Institute (AHRI) in Addis Abeba in February 2009. A total of 39 (12 + 12 + 3 +12) volunteers were allocated to the following 4 groups:

group 1: TST/QF negative (receiving H1 alone)
group 2: TST/QF negative (receiving H1 + IC31)
group 3: BCG vaccinated (receiving H1 + IC31)
group 4: Latent TB (receiving H1 + IC31)

This trial is still awaiting release of the independent assessor's report and immunogenicity data. Preliminary results show that adverse events from this trial were similar to those of the two concluded trials: all adverse events were mild apart from two possibly vaccine-related

serious adverse events in otherwise clinically stable participants which resolved in less than 72 hours were reported as follows:

- Increased ASAT/ALAT, fever, chills, generalized body weakness and erythema at previous PPD injection site 12 hours post vaccination.
- CPK elevated to 10545 IU/L 24 hours post 1st vaccination and 11025 IU/L 48 hours post 1st vaccination. This occurred in a heavy weight lifter which might explain the increase in CPK.

Two clinically stable volunteers were withdrawn after experiencing mild adverse events that were considered to be possibly related to the vaccine. One had progressively increasing eosinophilia stable, and the other had erythema and itching at his previous PPD injection site.

2. Trial objectives

2.1 Primary objectives

To evaluate the safety of H1/IC31®, an adjuvanted TB subunit vaccine administered to HIV-infected adult subjects with no evidence of active TB disease.

To determine the immunogenicity of H1/IC31® in HIV-infected adult subjects with no evidence of TB disease.

2.2 Secondary objectives

To describe the effect of the H1/IC31® TB vaccine in HIV-infected adults on:

- CD4+ lymphocyte counts
- HIV viral loads

2.3 Exploratory objectives

To evaluate innate and adaptive immune response to H1/IC31® in HIV-infected adults using transcriptomics, multi-colour flow cytometry, multi-plex luminex assays and quantitative real time PCR.

2.4 Primary variables

The primary variables will be based on medical examinations, blood and urine samples, and self reporting, and comprise:

A. Physical examinations, including ENT, cardiovascular, pulmonary, neurological, gastrointestinal, urogenital, and dermatological systems, as well as injection sites.

B. Local adverse events, including pain, erythema, induration, swelling, itching, nodules, and local functional limitations.

C. Systemic adverse events, including diffuse erythema, urticaria, asthma, angioedema, fatigue, fever, arthralgia, myalgia, hoarseness, dizziness, malaise, paleness, sweating, nausea, arrhythmia, headache, and anaphylaxis.

2.4.1 Laboratory safety tests:

A. Blood samples will be tested for RBC, differential WBC, platelets, haemoglobin, haematocrit, ASAT, ALAT, alkaline phosphatase, lactate dehydrogenase, γGT, albumin, bilirubin, creatinine, glucose.

B. Urine samples will be tested for glucose, protein and sediment.

2.4.2 Immunogenicity variables:

A. IFNγ spot-forming cells in PBMCs after stimulation with Ag85B peptides, ESAT-6 peptides and H1 protein detected by ELISPOT.

B. IFNγ production in supernatants of PBMC stimulated with Ag85B peptides, ESAT-6 peptides and H1 protein detected by ELISA.

C. Frequency and magnitude of type-1 cytokines in CD4 and CD8 T cells after short-term stimulation of whole blood with Ag85B peptides, ESAT-6 peptides, H1 protein, and BCG by intracellular cytokine staining (ICS) before and after vaccination with H1/IC31.

D. IgG antibodies to H1 in serum detected by ELISA.

2.5 Secondary variables

The secondary variables will be based on HIV related laboratory assessments:

A. Detection of CD4 counts by flow cytometry.

B. Detection of HIV-1 viral load by Roche amplicor.

2.6 Exploratory variables

- Frequencies of activated and proliferating T cells by analysing Ki67 nuclear protein expression.
- Frequency and phenotype of the central memory T cells responses in PBMCs stimulated with Ag85B peptides, ESAT-6 peptides, H1 protein, and BCGmeasured using multi-colour flow cytometry.
- Cytokine and chemokine mRNA expression in PBMCs stimulated with Ag85B peptides, ESAT-6 peptides, H1 protein, and BCG measured by quantitative real time PCR in cell pellets.
- Cytokines and chemokine proteins secreted by PBMC after Ag85B peptides, ESAT-6 peptides, H1 protein, and BCG stimulation measured by multiplex luminex technology in culture supernatant.
- Frequency and magnitudes of type-1 cytokines in CD4 and CD8 T cells after short-term stimulation of PBMCs with Ag85B peptides, ESAT-6 peptides, H1 protein, and BCG by ICS before and after vaccination with H1/IC31.
- Profile of transcriptional gene expression observed in whole blood collected at day 3 and 59 versus the day 0 baseline.

3. Investigational plan

3.1 Overall design

This is a Phase II, randomized, double-blind, placebo-controlled trial evaluating the safety and immunogenicity of H1/IC31® administered to HIV-infected adult subjects with no evidence of TB disease.

Randomization will be performed on Study Day 0 prior to the administration of the study vaccine. Subjects will be randomly allocated in a 5:1 ratio to receive either H1/IC31® vaccination or placebo, administered on Study Days 0 and 56.

3.2 Study population

The study will be conducted at clinical trial sites in Bagamoyo District, Tanzania and Ekurhuleni District, Johannesburg, South Africa. One study group is planned, with an anticipated enrolment of 48 subjects split evenly between the two sites (see Table 1).

Table 1: Summary of study groups

Site	H1 /IC31® Dose volume	Number of Subjects		
		H1/IC31®	Placebo	Total
Bagamoyo	0.5ml	20	4	24
Johannesburg	0.5ml	20	4	24
**TOTAL**		**40**	**8**	**48**

3.3 Recruitment and Informed consent

Subjects will be recruited from referrals from local clinics, contacts made by recruiters in the community or solicitation of subjects previously known to the clinical trial sites. Interested subjects will be invited to participate in the informed consent process. Informed consent will be obtained by the use of a written or witnessed verbal consent form approved by the IRB/IEC and signed and dated by the subject at the time of consent. The clinical investigator will conduct the consent discussion with each subject and allow adequate time for all questions to be addressed.

A copy of the signed consent form will be given to the subject prior to randomization.

3.4 Inclusion criteria

1. Age between 18 and 55 years
2. Has laboratory evidence of HIV infection, defined as a positive HIV-1 ELISA or rapid ELISA test plus a positive confirmatory test (e.g., a second HIV-1 ELISA, PCR, or rapid ELISA)
3. Have two CD4+ lymphocyte counts, at least four days apart within the screening period, greater than 350 cells / mm^3
4. Not currently receiving antiretroviral drugs
5. Healthy based on medical examination or history at inclusion
6. Females: Ability to avoid pregnancy during the trial: Women physically capable of pregnancy (not sterilized and still menstruating or within 1 year of the last menses if menopausal) must avoid pregnancy from 21 days prior to administration of the study vaccine through the end of the study. Acceptable methods of avoiding pregnancy include a sterile sexual partner, sexual abstinence (not engaging in sexual intercourse), or use of a combination of at least two forms of acceptable contraception: hormonal contraceptives (oral, injection, transdermal patch, or implant), vaginal ring, intrauterine device (IUD), and the use of a condom or a diaphragm; or the use of a condom or a diaphragm combined with spermicide.
7. Is able to carry out activities of daily living independently
8. Is able and willing to comply with study procedures and to complete the follow-up period as required by the protocol
9. Is able and willing to commit to avoiding elective surgery for the duration of the study
10. Is prepared to grant authorized persons access to the medical records
11. Has provided signed informed consent

For a volunteer to be included in the trial, all of the criteria listed above must be answered with a “yes”.

3.5 Exclusion criteria

1. Acute illness
2. Fever ≥37.5°C at enrolment
3. Evidence of or suspected active TB
4. Used immunosuppressive medication within 42 days prior to randomization (inhaled and topical corticosteroids are permitted.)
5. Received immunoglobulin or blood products within 42 days prior to randomization
6. Medical history that in the opinion of the investigator may compromise the evaluation of safety of the subject in the study (e.g., diabetes, seizure disorder, sickle cell disease)
7. Pregnant or breastfeeding female, or intending to become pregnant during the study period
8. Received any investigational drug therapy or vaccine within 182 days prior to randomization
9. Known hypersensitivity to any of the vaccine components
10. Laboratory parameters outside of normal range considered clinically relevant

3.6 Table 2. Schedule of events

	**Study day**	**Screening**		**0**	**3**	**14**	**54**	**56**	**59**	**70**	**182**
	Visit window (days)	-28 to 0		-	± 1	± 2	± 1	± 1	± 2	± 2	± 2
	Visit number	1	2	3	4	5	6	7	8	9	10
	Study Procedures										
	Locator information	X		X				X		X	
	Informed consent	X									
	Medical history	X									
	Physical examination	FM		AM	AM	AM	FM	AM	AM	AM	FM
	AEs & con meds	X	X	X	X	X	X	X	X	X	X
	Check for TB & OIs	X	X	X	X	X	X	X	X	X	X
	Pregnancy prevention	X	X	X	X	X	X	X	X	X	X
	Eligibility criteria	X		X				X			
	Chest X-ray	X									
	Screening Laboratory Assessments										
	HIV rapid tests	X$^{(5)}$									
	QGIT	X$^{(3)}$ [a]									
	Safety Laboratory Assessments										
	WBC, differential, platelets [b]	X$^{(5)}$ [d]				X$^{(5)}$	X$^{(5)}$ [d]			X$^{(5)}$	X$^{(5)}$
	Serum chemistry [c]	X$^{(5)}$ [d]				X$^{(5)}$	X$^{(5)}$ [d]			X$^{(5)}$	X$^{(5)}$
	Urine βHCG	X[e]		X[e]				X[e]			
	Urinalysis	X		X	X	X	X	X	X	X	X
	CD4+ count	X$^{(2)}$	X$^{(2)}$	X$^{(2)}$		X$^{(2)}$		X$^{(2)}$		X$^{(2)}$	X$^{(2)}$
	HIV-1 viral load	X$^{(2)}$		X$^{(2)}$		X$^{(2)}$		X$^{(2)}$		X$^{(2)}$	X$^{(2)}$
	Immunogenicity Laboratory Assessments										
PBMCs	ELISpot & ELISA			X$^{(8)}$		X$^{(8)}$		X$^{(8)}$		X$^{(8)}$	X$^{(8)}$
	PBMC ICS			X$^{(8)}$		X$^{(8)}$		X$^{(8)}$		X$^{(8)}$	X$^{(8)}$
	PCR and luminex after stimulation			X$^{(8)}$		X$^{(8)}$		X$^{(8)}$		X$^{(8)}$	X$^{(8)}$
	Proliferation assays			X$^{(8)}$		X$^{(8)}$		X$^{(8)}$		X$^{(8)}$	X$^{(8)}$
	Specimen storage			X$^{(16)}$		X$^{(16)}$		X$^{(16)}$		X$^{(16)}$	X$^{(16)}$
WB	WBA ICS<br>Ex vivo Ki67<br>IgG assay			X$^{(10)}$		X$^{(10)}$		X$^{(10)}$		X$^{(10)}$	X$^{(10)}$
RNA	Transcriptomics			X$^{(2.5)}$	X$^{(2.5)}$				X$^{(2.5)}$		
	Vaccination procedures										
	Vaccination			X				X			
	Reactinogenicity			X	X	X		X	X	X	
	Injection site exam			X	X	X		X	X	X	
	Blood mL collected per visit [f]	22	2	64.5	2.5	72	10	62	2.5	72	72
	Cumulative blood mL collected [f]	22	24	88.5	91	163	173	235	237.5	309.5	381.5

FM=Full medical examination; AM=Abbreviated medical examination; OIs=Opportunistic infections; QGIT=Quantiferon Gold In-tube assay; AEs=adverse events; ICS= intracellular cytokine staining; WBA=Whole Blood Assay; WB=Whole Blood; Con Meds=Concomitant medications, Hep=Hepatitis.

(Numbers) represent the volume of blood drawn for the investigation.

a. Screening safety laboratory investigations are for the purpose of establishing baseline values and are not considered exclusionary.

b. Safety: RBC, differential WBC, platelets, haemoglobin, haematocrit,

c. Safety: ASAT, ALAT, alkaline phosphatase, lactate dehydrogenase, γGT, albumin, bilirubin, creatinine, glucose.

d. Results of chemistry and haematology tests must demonstrate no adverse change in severity per the grading scales of the DAIDS Toxicity Table (Appendix 3) prior to vaccination on Study Day 56.

e. Urine pregnancy test must be performed and results confirmed (documented) to be negative prior to vaccination on Study Days 0 and 56.

f. Blood volumes are estimates. Specimens for all laboratory analyses will be collected according to local laboratory protocols except for immunology specimens which will be collected according to instructions provided by SSI.

3.7 Details of visits

Visit 1: First Screening Visit (Day -28 to -4)

- Allocate screening number
- Record locator information and contact details for at least 3 different means of contacting the participant.
- Establish a medical record, take records of demography, medical history and perform a full extended medical examination, including checking for symptoms of TB and opportunistic infections and document all baseline signs and symptoms.
- For female participants, confirm whether adequate methods to prevent pregnancy have been used for at least the past 21 days and provide pregnancy prevention counselling. If not, advise the participant accordingly.
- Obtain details of any medication taken by the participant within 42 days of study screening.
- Assess the volunteer according to the inclusion/exclusion criteria.
- If enrolment criteria are met on history and physical examination:
    - send the volunteer for a chest X-ray.
    - draw blood samples for screening (HIV rapid tests and QuantiFERON®-TB Gold test) and safety (FBC and differential, platelets, serum chemistry, HIV viral load, CD4+ count) tests.
    - collect a urine sample for urinalysis and pregnancy test.

Visit 2: Second Screening Visit (Day -10 to 0)

- For female participants, confirm whether adequate methods are being followed to avoid pregnancy. If not, defer enrolment and advise the participant accordingly.
- Document any additional baseline signs and symptoms.
- Check for symptoms of TB or opportunistic infections.
- If required, draw blood for second CD4+ count.

Visit 3: Baseline Visit (Day 0)

- Confirm locator and contact details for at least 3 independent means of contacting the participant are still valid.
- Conduct an abbreviated medical examination.
- Record signs of TB or opportunistic infections before the vaccination, which will form part of baseline signs and symptoms.
- Record use of concomitant medications.
- For female participants, confirm whether adequate methods to prevent pregnancy have been taken for ≥21 days.
- Draw blood for safety assessment before the vaccination.

- Collect urine for urine pregnancy test and urinalysis.
- Draw a blood sample for immunogenicity assessment and specimen storage.
- Confirm all eligibility criteria are met and that the pregnancy test is negative.
- Allocate the next available study number.
- Administer the first vaccination.
- Conduct reactinogenicity assessment and examine the injection site.

Visit 4: Follow-up Visit (Day 3 ±1)

- Conduct an abbreviated medical examination
- Conduct reactinogenicity assessment and examine the injection site.
- Record adverse events and signs of TB or opportunistic infections.
- Record use of concomitant medications.
- For female participants, confirm whether adequate methods are being followed to avoid pregnancy and provide pregnancy prevention counselling.
- Draw a blood sample for immunogenicity assessment and specimen storage.
- Collect urine for urinalysis.

Visit 5: Follow-up Visit (Day 14 ±2)

- Conduct an abbreviated medical examination.
- Conduct reactinogenicity assessment and examine the injection site.
- Draw a blood sample for immunogenicity assessment and specimen storage.
- Collect urine for urinalysis
- Record adverse events and signs of TB or opportunistic infections.
- Record use of concomitant medications.
- For female participants, confirm whether adequate methods are being followed to avoid pregnancy and provide pregnancy prevention counselling.

Visit 6: Follow-up visit (Day 54 ±1)

- Conduct a full medical examination.
- Record adverse events and signs of TB or opportunistic infections.
- Record use of concomitant medications.
- For female participants, confirm whether adequate methods are being followed to avoid pregnancy and provide pregnancy prevention counselling.
- Draw blood for safety tests.
- Collect urine for urinalysis.

Visit 7: Second Vaccination Visit (Day 56 ±2)

- Confirm locator and contact details for at least 3 independent means of contacting the participant are still valid.
- Conduct an abbreviated medical examination.
- Record adverse events and signs of TB or opportunistic infections.
- Record use of concomitant medications.
- For female participants, confirm whether adequate methods are being followed to avoid pregnancy and provide pregnancy prevention counselling.
- Draw blood for safety assessment.
- Collect urine for urine pregnancy test and urinalysis.
- Draw a blood sample for immunogenicity assessment and specimen storage.
- Confirm all eligibility criteria are still met and that the pregnancy test is negative.
- Administer the second vaccination.
- Do reactinogenicity assessment and examine the injection site.

Visit 8: Follow-up Visit (Day 59 ±2)

- Conduct an abbreviated medical examination.
- Conduct reactinogenicity assessment and examine the injection site.
- Record adverse events, signs of TB or opportunistic infections.
- For female participants, confirm whether adequate methods are being followed to avoid pregnancy and provide pregnancy prevention counselling.
- Draw blood for immunogenicity tests and specimen storage.
- Collect urine for urinalysis.

Visit 9: Follow-up Visit (Day 70 ±2)

- Confirm locator and contact details for at least 3 independent means of contacting the participant are still valid.
- Conduct an abbreviated medical examination.
- Conduct reactinogenicity assessment and examine the injection site.
- Record adverse events and signs of TB or opportunistic infections.
- Record use of concomitant medications.
- For female participants, confirm whether adequate methods are being followed to avoid pregnancy and provide pregnancy prevention counselling.
- Draw a blood sample for immunogenicity assessment and specimen storage.
- Collect urine for urinalysis.

Visit 10: Follow-up Visit (Day 182 ±2)

- Conduct a full medical examination.
- Record adverse events and signs of TB or opportunistic infections.
- Record use of concomitant medications.
- Draw a blood sample for immunogenicity assessment and specimen storage.
- Collect urine for urinalysis.
-

3.8 Handling of blood samples

3.8.1 Sample collection

Venous blood samples will be drawn according to GCP guidelines by experienced medical personnel (for schedule see flowchart): for the QuantiFERON®-TB Gold test, to obtain serum, for blood count and clinical chemistry and isolation of viable cells and samples to be tested for HIV.

The following tubes will be used:

- Serum separation tubes (SST) for chemistry
- Ethyldiamine tetra acetic acid (EDTA) tubes FBC, CD4 counts, viral loads and plasma storage
- Cell preparation Tubes (CPT) with heparin for isolation of viable cells (PBMC)
- QuantiFERON®-TB Gold test tubes
- Paxgene tubes (transcriptomics)
- Which tubes will be used to obtain the plasma for ELISA assays from the CPT tubes

3.8.2 PBMC isolation and storage

PBMCs will be isolated from whole blood collected in cell preparation tubes (CPT) according to instructions provided by BD Biosciences. Isolated PBMCs will be decanted into several vials and frozen in 10% DMSO in fetal bovine serum freezing media. The cryopreserved PBMCs will be transported to the various laboratories conducting the analyses using PBMCs listed in section 3.9.

3.9 Laboratory immunogenicity analyses

Several immunological analyses will be performed to assess the immune responses induced by the H1/IC31® vaccine. The various tests will use peripheral blood mononucleocytes (PMBCs), whole blood, serum or RNA isolated from whole blood. All samples for immunological assessments will be processed shortly after being collected, then cryopreserved until the end of the study when the samples will be distributed to the central labs for each assessment.

The primary objective of the study, to determine the immunogenicity of H1/IC31®, will be assessed using ELISpot, ELISA and 12-hour ICS in a whole blood assay (WBA). Exploratory assessments will be conducted to characterise the cellular mechanisms involved in the immunogenic response to the vaccine by performing: ICS and PCR assessments following fresh PBMC stimulation; Ki67 protein expression analyses; chemokine and cytokine mRNA

and cDNA expression analyses; phenotypic determination of proliferating T cells; and transcriptomic analyses. A description of each assay is provided in the following sections.

3.9.1 ELISpot and ELISA

These assays will utilize either fresh or frozen PBMCs. For ELISpot and ELISA assays utilizing freshly isolated PBMC, stimulation and measurement of immune responses will be completed soonest after blood collection. For Elispot and Elisa assays utilizing frozen PBMCs, the cells will be thawed, then stimulated with recombinant H1 and overlapping Ag85B and ESAT6 peptide pools. PBMCs stimulated with phytohemagglutinin (PHA) will be used as positive controls and un-stimulated PBMCs will be used as negative controls.

Frozen PBMCs will be thawed and incubated at 37°C overnight in the presence of relevant antigens. The following day stimulated PBMCs will be analyzed by ELISPOT for the number IFNγ spot-forming cells.

Frozen PBMCs will be thawed and incubated at 37° Celsius for 7 days in the presence of relevant antigens. Subsequently, the supernatants are analyzed by ELISA for IFNγ production.

Frozen serum will be thawed and analyzed by ELISA for presence of antibodies specific to recombinant protein H1.

3.9.2 Quantitative real time PCR analysis of PBMC after stimulation

Detection of the vaccine antigen specific induction of chemokines and cytokines in vitro will be conducted to provide a detailed characterization of the quality and quantity of the cellular immune responses induced. Freshly isolated PBMCs will be incubated for 12h in the presence of the recombinant H1, overlapping peptide pools of Ag85B, ESAT6, BCG, PHA or nothing (negative control). Cell pellets will be collected and stored in RLT buffer (Qiagen) at -80ºC to maximize the stability of the RNA. The samples will be transported at the end of the study to the centralized facility at the Swiss Tropical and Public Health Institute, Basel, Switzerland where the total RNA and cDNA will be prepared and followed by quantitative real time PCR based assessment of Th1 and Th2 signature cytokines and chemokines.

3.9.3 Multi-plex quantification of cytokine and chemokines in PBMC after stimulation

Secreted cytokines after vaccine antigen stimulation in vitro will allow for characterization of the quality of the cellular immunity induced. Culture supernatants of the PBMC cultured under 3.9.2 with the above conditions will be collected and stored at -80ºC for analysis using microsphere suspension array technology at the end of the study. This will take place at the Swiss Tropical and Public Health Institute, Basel, Switzerland.

3.9.4 Analysis of intracellular cytokines by multi-colour flow cytometry after stimulation

Detection of the phenotype of cytokine producing, vaccine specific T cells will allow for assignment of the cytokine production to multiple vs single cytokine producing T cells. PBMC cultured with recombinant H1, overlapping peptide pools of Ag85B, ESAT6, BCG, PHA and negative control will be analyzed by ICS and multi-colour flow cytometry in Bagamoyo.

Cytokines analyzed will include INF-g, TNF-a and IL-2 while the lineage markers analyzed will be CD4, CD8, CD3.

3.9.5 Proliferation assay of effector central memory cellular immunity after stimulation

The phenotype of proliferating vaccine specific T cells will be detected to assess the central memory T cells induced by vaccination. Carboxyfluorescein diacetate succinimidyl ester (CFSE) diffuses into the cytoplasm of cells, where it is cleaved by intracellular esterases to yield fluorescent compounds. These compounds bind to amino groups of intracellular proteins and remain in the cell with considerable stability. The amount of fluorescent compound is proportioned equally between daughter cells during mitosis and hence decreases by half at each division.

Purified PBMCs will be labelled with CFSE and cultured in the presence of recombinant H1, overlapping peptide pools of Ag85B, ESAT6, BCG, PHA and negative control for 6 days. Cells will be harvested and further stained with fluorochrome-labelled antibodies specific for CD3, CD4, CD8 and cell dead dye. Multicolour flow cytometry will be used to simultaneously identify the phenotype and proliferation status of CFSE-labelled cells.

3.9.6 Whole blood intracellular cytokine staining assay

Whole blood ICS assay will be performed for six conditions: H1, Ag85B, ESAT-6, BCG, PHA and Nil (control). Each condition will be analysed using 1mL whole blood (total of 6mL whole blood). Whole blood will be added to pre-prepared tubes containing the individual antigens (recombinant H1, overlapping peptide pools of Ag85B and ESAT6, BCG, PHA) and co-stimulatory antibodies (anti-CD28 and anti-CD49d). The whole blood will be incubated at 37°C for 12 hours. After 7 hours of the incubation, 200μL (2 aliquots each of 100μL) of plasma will be replaced with Brefeldin-A. The blood will be harvested with EDTA, red blood cells lysed and white cells fixed with FACS lysing solution. The white cells will be pelleted followed by cryopreservation with a freezing media for later ICS. A multicolour flow cytometry ICS panel will be optimized to measure vaccine-induced immune responses.

3.9.7 Ex-vivo Ki67 assay

The hallmark of a vaccine-mediated protection is the induction of an effective immunological memory. Following vaccination, B and T cells specific to the vaccine expand (expansion phase) and carry out the effector functions. Following resolution (contraction phase) of the vaccine induced response; the immune system maintains a population of B and T cells (central memory) that is specific to the vaccine.

Ex-vivo Ki67 expression will be used as an indicator of cell proliferation soon after vaccination to identify expanding populations of activated T cells, unlike the other immunological assays being conducted which will be measuring the central memory component of vaccine-induced immunity.

Whole blood (200μL) drawn on the day of vaccination, two weeks after each vaccination and the last day of follow-up will be lysed with FACS lysing solution. The white cells will be pelleted and cryopreserved in 10% DMSO in fetal bovine serum then transported to the central laboratory that will perform the Ki67 ICS.

3.9.8 IgG assay

Presence of antibodies specific to mycobacteria antigens in individuals exposed to *M.tubercolosis* are thought to be important in mediating protection against infection or development of TB disease. In this assay, we will measure IgG antibodies present in serum or plasma collected before and after H1/IC31® vaccination. At each time point of immunogenicity assessment, 2mLs of whole blood will be collected in blood collection tubes with clot activators. The blood will be allowed to stand at room temperature until a clot forms then centrifuged for 15minutes. Serum will be collected and frozen in three aliquots for later IgG assessment by ELISA.

3.9.9 Transcriptomics

Genome-wide studies of gene expression levels will be performed to quantify the variation and presence of mRNA following vaccination. In this assay, 2.5mL whole blood is pipetted into a PAXgene blood RNA tube followed by incubation for 2hrs at room temperature. The processing of the samples will be done as per the manufacturer's (PreAnalytiX Qiagen) recommendation. The PAXgene Blood RNA Kit will be used for cellular RNA extraction. Two aliquots (40µL each) of RNA extracts will be stored at -200C for later analysis.

3.10 Time Schedule

The first visit for the first volunteer is expected to take place in Q3 2011, and last volunteer's last visit is expected to take place in Q3 2012.

3.11 Withdrawal and predetermined reasons for discontinuation

Volunteers are free to withdraw from the trial whenever they desire. If, for any reason, a volunteer wishes to discontinue his participation in the trial, or if, according to sponsor's or investigator's judgment, he must be withdrawn from the trial, the date and reason for the withdrawal are to be recorded in the CRF. Data from volunteers that are withdrawn from or discontinue the trial will be included in the final report. Volunteers that are withdrawn from or discontinue the trial will not be replaced. If the following symptoms are recorded within 3 hours after vaccination, the volunteer must be withdrawn from the trial: Suspected immediate or delayed allergic or anaphylactic reaction.

The trial can be terminated at any time if the sponsor or investigator determines that the trial poses a serious threat to the volunteers, taking into account the DSMB recommendations.

3.12 Pregnancy

If a female participant becomes pregnant during the course of the study and this is verified by a positive Urine βHCG urine test, the participant will not receive any further vaccination.

To assess any adverse events, the investigator will follow up the participant until the end of the pregnancy. The child will be further followed up for at least 16 weeks after birth. The Sponsor will request access to medical records of the mother and the child from the start of pregnancy until at least 16 weeks after birth of the child.

4. Investigational product

4.1 Treatments administered

H1/IC31® is an experimental adjuvanted TB vaccine supplied as a sterile suspension for injection with a pH of 7.4. The IC31®adjuvant and the final vaccine product are manufactured by SSI.

The concentration of the active pharmaceutical ingredients and buffer in the investigational products are as follows:

**Vaccine product**	
Antigen	50 µg
IC31 adjuvant:	
KLK	500 nmol/ml
ODN1a	20 nmol/ml
Buffer system	Trometamol 10 mmol/l<br>Sodium chloride<br>135 mmol/l
**Placebo**	
Tris Buffer	Trometamol 10 mmol/l<br>Sodium chloride<br>135 mmol/l

4.2 Packing and labelling

The investigational products will be supplied by SSI with each vial individually packed.

Label for the box:

Trial: Aurum 102/THYB-05 Participant No:_____
Statens Serum Institut,<br>Artillerivej 5 2300 DK<br>1 dose = 0.5 ml i.m.<br>Suspension for injection<br>Investigator:<br>Name of site:<br>Telephone:
Shake before use<br>**For clinical trial use only**
Batch:<br>Storage: at < -15ºC
Expiry date:

Label for the vials:

Trial: Aurum 102/THYB-05 Participant No:_____
50 µg antigen<br>1 dose = 0.5 ml.<br>Investigator:<br>Batch:

4.3 Doses and administration

A volume of 0.5 ml will be administered which will provide a dose of 50 µg Ag85B-ESAT6 (antigen) and 500 nmol KLK + 20 nmol ODN1a (adjuvant) or Tris buffer (placebo). Each vial contains 1 dose of 0.5 ml with a surplus of approximately 0.3 ml.

The vaccine or placebo must be shaken vigorously immediately before administration. After shaking, the vaccine is a slightly turbid white to off-white suspension.

The vaccine will be administered intramuscularly, the first injection into the right and the second into the left deltoid. The vaccination site should be disinfected with a skin disinfectant (for example, 70% alcohol) and allowed to dry before vaccination. A 22 to 25-gauge needle should be used and the needle length should be 2.5 to 4 cm.

4.4 Vaccine storage and dose preparation

The shelf life of the vaccine is 48 months and 36 months for the placebo. Expiry dates will be indicated on the box labels. The investigational products must not be used after the expiry dates.

The investigational products must be stored secured in the site pharmacy at a temperature below -15°C and protected from light. Investigational products that have been exposed to temperatures outside this temperature range must not be used.

Before use, the investigational products must be thawed at room temperature for at least 45 minutes. Once thawed the vaccine must be used within 5 hours. The person administering the vaccine must confirm the filled syringe does not feel cold prior to administration.

The vaccine or placebo must be shaken vigorously immediately before administration.

4.5 Transport of trial products

The investigational products will be packed at SSI and sent directly to the site via courier. Temperature loggers will be packed with the investigational products to monitor the temperature during transport from SSI to the storage facilities at the trial site. When the boxes are unpacked, the temperature loggers will be sent back to SSI who will download the temperature data and confirm the investigational product can be used before any product is administered.

4.6 Treatment Group Assignment or Randomization

Once it has been confirmed that a subject still meets the enrolment criteria, the next sequential subject number will be allocated. The unique subject identification number will link the subject to a treatment assignment of either H1/IC31® vaccine product or placebo. The pharmacist will prepare the vaccination according the pre-prepared study randomisation list.

If a subject does not meet all study entry criteria and the situation permits, subjects may be reconsidered for randomization at a later date following resolution of an acute illness or after a repeated clinical laboratory evaluation result is shown to be within normal limits, provided the allowed time frame for screenings is met.

4.7 Replacement of withdrawn subjects

Once the first dose of investigational product has been administered, the subject will be considered enrolled, and cannot be replaced.

4.8 Study Blind

The appearance of the prepared doses of H1/IC31® and placebo vary slightly after shaking, so to maintain the blind, syringes will be wrapped in opaque tape once filled.

The pharmacist and study monitors will be the only unblinded study personnel.

All pharmacy records must be kept in a secure location with access strictly limited to the study pharmacist until the clinical site is notified by SSI or its designee that the study has been unblinded.

4.9 Unblinding for Clinical Emergencies

There will be one set of emergency envelopes at the trial site kept under the responsibility of the principal investigator, and there will be one set in the Department of Regulatory & Medical Affairs, SSI, kept under the responsibility of the Quality Person for Pharmacovigilance (QPPV), SSI.

In the event of a medical emergency, the code may be broken by the investigator ONLY if the information given in the emergency code envelope is of relevance for the further treatment of the volunteer. In this case, the code must be kept STRICTLY CONFIDENTIAL, and must

ONLY be revealed to research staff directly involved in the medical emergency on a need to know basis.

The Department of Regulatory & Medical Affairs, SSI may break the code ONLY if necessary to comply with serious adverse reaction reporting requirements of the authorities. In this case, the code must be kept STRICTLY CONFIDENTIAL and must under no circumstances be revealed to any person not directly involved in the reporting, including the principal investigator, the clinical trial manager, the trial statistician or any person working with laboratory or statistical analysis of the trial data and interpretation of the trial results.

If the code is broken, the time, date and reason for breaking it must be recorded on the emergency envelope together with the signature of the person breaking it. The broken emergency code envelope must be kept together with the volunteer's case record form if broken at the trial site, or according to internal procedures at SSI if broken in the Department & Regulatory & Medical Affairs, SSI.

The clinical trial manager at SSI must be informed within 72 hours after breaking the code/envelope (without being informed about the actual treatment code).

4.10 Treatment compliance, drug accountability

The investigational product must be kept in a safe place during the trial under supervision of the investigator. Full accountability records and storage temperature logs must be maintained by the study pharmacist as long as product is stored at the site.

All used vials must be stored in their original boxes until the monitor has verified the accountability records. SSI will give instructions at the end of the study whether unused investigational product should be returned to SSI or destroyed.

4.11 Precautions

Anaphylactic reactions are a potential risk, and should be managed according to local standard of care. Adrenaline and a resuscitation kit with instructions for use should be kept available for immediate use in the room where the injections are administered.

4.12 Concomitant medication

Permitted concomitant medication considered necessary for the medical management of subjects during the trial must be documented including the drug, dosage, route of administration, indication and start and stop dates.

4.13 Prohibited medication

All systemic immunosuppressant medications are prohibited throughout the study.

5. Adverse events

5.1 Definitions and terms

All definitions are according to the ICH E2A guidelines.

5.2 Adverse event

Any untoward medical occurrence experienced by a volunteer participating in a clinical investigation and receiving a pharmaceutical product, which does not necessarily have a causal relationship with this product.

5.3 Adverse reaction or suspected adverse reaction

Any untoward and unintended response to an investigational product related to any dose administered. The terms ‘Adverse Reaction’ and ‘Adverse Drug Reaction are the same thing (in practice), and imply that there is a suspected relationship between the event and the trial product. In practice this means that there is evidence or arguments that suggest a causal relationship, i.e. a relationship cannot be ruled out.

For further details, see ICH Topic 2A and EU directive 2001/20/EC.

5.4 Unexpected Adverse Drug Reaction

An adverse reaction, the nature or severity of which is not consistent with the applicable product information (e.g., Investigator's Brochure for an unapproved investigational medicinal product).

To ensure no confusion or misunderstanding of the difference between the terms "serious" and "severe," which are not synonymous, the following note of clarification is provided:

The term "severe" is often used to describe the intensity (severity) of a specific event (as in mild, moderate, or severe myocardial infarction); the event itself, however, may be of relatively minor medical significance (such as severe headache). This is not the same as "serious," which is based on patient/event outcome or action criteria usually associated with events that pose a threat to a patient's life or functioning. Seriousness (not severity) serves as a guide for defining regulatory reporting obligations.

5.5 Seriousness criteria

A serious AE or AR is any untoward medical occurrence that at any dose:

- results in death
- is life-threatening
- requires hospitalisation or prolongation of hospitalisation
- results in persistent or significant disability/incapacity
- is an important medical condition

NOTE: The term ‘life-threatening’ in the above definition refers to an event during which the volunteer was at risk of death at the time of the event, it does not refer to an event which hypothetically could have caused death, had it been more severe.

For further details, see ICH Topic 2A and EU directive 2001/20/EC.

5.6 Unexpected adverse reaction

An ADR, the nature or severity of which is not consistent with the applicable product information (e.g., the Investigator’s Brochure).

If a serious suspected adverse reaction is unexpected it is a ‘Suspected Unexpected Serious Adverse Reaction’ (SUSAR).

For further details, see ICH Topic 2A and EU directive 2001/20/EC.

The expectedness of non-serious ADR will be determined during the final analysis of the data collected in the case report forms.

5.7 Documentation of adverse events

The investigator is responsible for the recording of all relevant information about adverse events using the following terms:

5.7.1 Causality

The causal relationship between the AE and the trial tests is assessed using the following terms:

- Not related
- Possibly related
- Probably related
- Definitely related

5.7.2 Severity

The intensity of an AE is assessed using the following terms:

- Mild (i.e. easily tolerated)
- Moderate (i.e. sufficient to interfere with daily activities)
- Severe (i.e. sufficient to prevent normal activity)

Furthermore, for AEs that are included in the National Institute for Health Division of AIDS (DAIDS) Toxicity Table (2004), the intensity will as far as possible be rated according to the more specific rating system suggested in this guidance document. If an AE is assessed as potentially life threatening (Grade 4), this implies that the AE is serious and that expedited reporting to SSI is required.

5.7.3 Outcome

The outcome of an AE is assessed using the following terms:

- Fatal
- Not yet recovered
- Recovered with sequelae
- Recovered without sequelae
- Unknown

NOTE: If an AE is still ongoing at the last visit, it must be followed by the investigator until it has resolved or stabilised.

5.7.4 Seriousness

The seriousness of an AE is assessed by answering the following questions:

- Did the event result in death?
- Has the event been or is the event life-threatening?
- Has the event required inpatient hospitalisation or prolongation of hospitalisation?
- Has the event resulted in significant or persistent disability or incapacity?
- Is the event medically important?

5.8 Reporting of adverse events

5.8.1 Reporting SAEs to SSI

The investigator is responsible for notifying SSI of any SAE as soon as possible but within 72 hours of any site staff becoming aware of the event by completing an adverse event report.

The completed report form must be sent to the Department of Regulatory & Medical Affairs at SSI.

The completed AE report from must be sent by fax to:

Statens Serum Institut

Department of Regulatory & Medical Affairs

Fax: +45 32 68 39 73

Attention: Medical Affairs

The initial report should be followed by follow-up reports (using the same form) if additional important information becomes available.

5.8.2 SSI's expedited reporting of SUSARs to the regulatory authorities and ethics committees

The timelines from SSI's first knowledge of an event that requires expedited reporting to reporting to the competent authorities and ethics committees for South Africa and Tanzania are given in Table 3 below.

Table 3. Adverse Event Reporting Timelines.

The number of calendar days from SSI's first knowledge of an adverse event until the day the expedited report must be submitted to the competent authorities in South Africa and Tanzania.

	South Africa	Tanzania
SUSAR resulting in death<br>Initial report<br>Follow up	7 days<br>15 days	24 hours<br>14 days
For SUSARs resulting in life-threatening conditions<br>Initial report<br>Follow up	7 days<br>15 days	14 days
Other SUSARs	15 days	14 days
Serious Adverse Event (SAE)	6 monthly report	14 days
Non-serious unexpected AEs	Final study report	Final study report

The Department of Regulatory & Medical Affairs, SSI will be responsible for reporting unblinded SUSAR reports by fax according to the above mentioned timelines to the competent authorities detailed below.

The Department of Regulatory & Medical Affairs, SSI will be responsible for distributing **blinded** copies of SUSAR reports according to the above timelines to the following parties:

- the principal investigator
- the clinical trial manager at SSI

The **blinded** SUSAR reports will be filed in the trial master file by SSI and in the investigator's file by the investigator/monitor.

South Africa

Medicines Control Council

Attention: Registrar of Medicines

Fax: +27 12 312 3106

Tel: +27 12 312 0295

Wits Health Research Ethics Committee

Attention: Safety Reporting

Fax: +27 11 274 9281

Tel: +27 11 274 9280

Tanzania

Tanzanian Food and Drug Administration

Attention: Director General

Fax: +255 22 2450793

Tel: +255 22 2450751

IHI Institutional Review Board

Attention: Chairperson

Fax: +255 22 2771714

Tel: +255 22 2774714

Email: irb@ihi.or.tz

Tanzania Medical Research Coordinating Committee (MRCC)

Attention: Director General

Fax: +255 22 2121360

Tel: +255 22 2121400

5.8.3 Safety reporting to the relevant ethics committees

The Department of Regulatory & Medical Affairs, SSI is responsible for submitting a 6-monthly progress reports in a line listing format of all unblinded suspected serious adverse reactions, including all SUSARs and SAEs to the Medicines Control Council and to the ethics committees. This will be facilitated by Aurum and Ifakara, via hand delivered courier with appropriated signed receipt.

Safety Reports should be prepared at 6-monthly intervals from the date of the CTA approval and be submitted within 60 days. A safety report should be prepared within 90 days of the end of the trial, together with the end of trial notification.

The Department of Regulatory & Medical Affairs, SSI will be responsible for distributing blinded copies of the safety reports to the principal investigator and the clinical trial manager at SSI.

The blinded SUSAR reports will be filed in the trial master file by SSI and in the investigator's file by the investigator/monitor.

6. Stopping Rules

The trial can be terminated at any time if the sponsor or an investigator determines that the trial poses a serious threat to the volunteers, taking into account the DSMB recommendations.

7. Data management and statistical analysis

7.1 Data management

The Data Management Department at Aurum will be responsible for data management and statistical analysis of all data from the trial. The data manager will be responsible for compiling a Data Management Plan, which will describe the methods used to collect, check and process the clinical data. The plan will also clarify the roles and responsibilities of the various functions and personnel involved in the data management process.

All data will be processed according to international standards including FDA CFR part 11 and ICH GCP. The data management team will be responsible for ensuring these guidelines are adhered to in order to provide a study database that is accurate, secure, reliable and ready for analysis.

7.2 Statistical analysis

An intent to treat and per protocol analyses will be conducted for all safety and immunogenicity parameters. The intent to treat study population is defined as participants who have received at least one vaccination.

Prior to analysis a detailed statistical analysis plan will be written which will include the definition of per protocol.

7.3 Primary endpoints

7.3.1 Adverse events:

All local adverse events and systemic adverse events will be listed by treatment group and visit.

Adverse events will be summarised by system organ class and preferred terms by treatment group and visit for each treatment group using descriptive statistics.

7.3.2 Laboratory safety tests:

Laboratory parameters will be listed as absolute values and change from baseline per treatment group and visit. Laboratory parameters will be summarised by treatment group and visit, for absolute and change from baseline using descriptive statistics (mean, standard deviation, median, minimum and maximum).

7.3.3 Immunogenicity variables:

Immunogenicity parameters will be listed as absolute values and change from baseline (visit 3) per treatment group and visit. Immunogenicity parameters will be summarised by treatment group and visit, for absolute and change from baseline using descriptive statistics (mean, standard deviation, median, minimum and maximum). Note that the primary immunogenicity timepoints are visit 5 and 9. Immunogenicity bloods are taken from visit 3-10 apart from visit 6.

7.4 Secondary endpoints

For CD4+ count and HIV-1 viral load the baseline measurement will be calculated as the arithmetic mean of visits 1,2,3 and visits 1 and 3, respectively.

7.5 Exploratory endpoints

Gene expression and transcriptional profile of blood collected at day 3 and 59 will be listed and summarised by treatment arm and visit, using descriptive statistics.

7.6 Sample Size

7.6.1 Sample size for the primary endpoints

The primary objective of this study is to evaluate the safety of H1/IC31®, an adjuvanted TB subunit vaccine administered to HIV-infected adult subjects with no evidence of active TB disease. Therefore, sample size calculation is based on safety and expressed in terms of the ability to detect adverse events (AEs) in follow-up. Specifically the sample size is based on the probability of observing a specified number of events based on the (i) total number of participants, (ii) AE incidence, and (iii) the follow-up time, assuming a Poisson distribution.

The scenarios are based on 40 participants, the AE incidence varying from 1to 15 per 100 person years and follow-up of 6 and 12 months.

Assuming a follow-up time of 6 months and an AE incidence of 10/100 person years, the probability of observing 1 or more AE is 0.87. If the AE incidence as high as 15/100 person years, the probability of observing 1 or more AE is 0.95.

Table 4. The probability of observing 0, 1 or more and 2 or more events under various scenarios.

#events	# individuals	rate/100 pys	FU time (years)	mean # events	Probability
0 events	40	1.0	1.00	0.4	0.670
1 or more	40	1.0	1.00	0.4	0.330
2 or more	40	1.0	1.00	0.4	0.062
0 events	40	3.5	1.00	1.4	0.247
1 or more	40	3.5	1.00	1.4	0.753
2 or more	40	3.5	1.00	1.4	0.408
0 events	40	5.0	1.00	2	0.135
1 or more	40	5.0	1.00	2	0.865
2 or more	40	5.0	1.00	2	0.594
0 events	40	10.0	1.00	4	0.018
1 or more	40	10.0	1.00	4	0.982
2 or more	40	10.0	1.00	4	0.908
0 events	40	1.0	0.50	0.2	0.819
1 or more	40	1.0	0.50	0.2	0.181
2 or more	40	1.0	0.50	0.2	0.018
0 events	40	3.5	0.50	0.7	0.497
1 or more	40	3.5	0.50	0.7	0.503
2 or more	40	3.5	0.50	0.7	0.156
0 events	40	5.0	0.50	1	0.368
1 or more	40	5.0	0.50	1	0.632
2 or more	40	5.0	0.50	1	0.264
0 events	40	10.0	0.50	2	0.135
1 or more	40	10.0	0.50	2	0.865
2 or more	40	10.0	0.50	2	0.594
0 events	40	15.0	0.50	3	0.050
1 or more	40	15.0	0.50	3	0.950
2 or more	40	15.0	0.50	3	0.801

pys = patient years; FU = follow up

7.6.2 Sample size for immunogenicity endpoints

The sample size calculations for the immunogenicity endpoint are based on the following assumptions :

- The response rate in the active arm is in the region of 70-90%.
- 90% power and a type I error of 5%.
- An approximate randomisation of active: placebo of 6:1.

Table 4. The probability of observing 0, 1 or more and 2 or more events under various scenarios.

Response in placebo arm	Response in active arm	Difference	# individuals in placebo arm	# individuals in active arm
10%	90%	80%	5	24
20%	90%	70%	6	33
30%	90%	60%	8	45
40%	90%	50%	11	63
5%	80%	75%	5	27
10%	80%	70%	6	34
15%	80%	65%	7	40
20%	80%	60%	9	49
5%	70%	65%	7	38
10%	70%	60%	9	47
15%	70%	55%	11	59

With 8 and 48 individuals in the placebo and active arms, respectively, there will 90% power to assess differences in the percentage responding assume response is between 70-90% in the active arm and the absolute difference is at least 60%.

(Note: in the THYB01 study the response rate was 100% using the vaccine regimen proposed here, after the 2nd vaccination).

8. Good Clinical Practice considerations

8.1 Applicable guidelines

The study will be conducted in accordance with the declaration of Helsinki, ICH E6 and pertinent local regulations under the auspices of local ethics committees, and institutional and national ethics committees where applicable.

8.2 Subject information and informed consent

Before entering the trial, volunteers must sign an informed consent form which complies with ICH E6 (Appendix 3). The volunteers will receive verbal and written information about the trial, including details of any potential risks.

8.3 Regulatory and ethics committee approval

The protocol, informed consent forms and all relevant associated documents must be approved by all required ethics committees before and study specific procedures are performed.

It is the responsibility of the investigator to file all correspondence associated with ethical approval and for informing the ethics committees of any SAE occurring during the trial and of any major amendments to the protocol. Major amendments to the protocol must be approved by the ethics committees prior to their implementation.

8.4 Subject data protection

The investigators are responsible for keeping a list (volunteer log) of all volunteers in the trial, including volunteer numbers, full names and last addresses known. The data management group will receive information about the volunteers' study number, sex and date of birth only. This information should, if necessary, be traceable to the volunteer log. The volunteers should be informed that the results will be stored and analysed by computer and that confidentiality will be maintained.

The volunteers should also be informed, both verbally and in writing that authorised persons, both from the sponsor and from the regulatory authorities, together with the investigator may review hospital records relevant for the trial.

Medical records and blood samples will be coded before they are sent for laboratory analyses, and only the investigator and his medical collaborators will have access to information that may link laboratory results with personal identification.

8.5 Amount of blood drawn

The total amount of blood required from each healthy volunteer throughout the trial period of 8 months will be approximately 364 ml which equals approximately 77% of a single blood donation (470 ml).

8.6 Compensation of volunteers and investigational institution

The volunteers will be reimbursed for any expenses incurred by participating in the study, and will be given a nominal amount to compensate for the time lost and inconvenience caused by volunteering for the study.

8.7 Investigators responsibilities

The Investigator is responsible for the conduct of this clinical trial in accordance with the protocol, current guidelines for Good Clinical Practice (GCP), and relevant local regulations. He/she undertakes this responsibility by signing the Investigator's Statement Form before study start (Appendix 1).

8.8 Indemnity statement:

Prior to the inclusion of the first subject in the clinical trial, SSI will present a signed Indemnity Statement to the investigators (Appendix 2).

8.9 Training

SSI will provide the Principal Investigators and other study personnel with relevant instructions and information needed for the conduct of this clinical trial in accordance with the current GCP guidelines and this Protocol.

The Principal Investigators are responsible for the adequate training of the study personnel involved.

8.10 Monitoring

Independent monitors will monitor the study on a regular basis throughout the study checking that the:

protocol is being followed

facilities and staffing remain acceptable

CRFs are being completed correctly

CRFs are in accordance with source data

clinical supplies (especially the trial vaccines) can be accounted for

serum and cell samples, and the vaccines are stored properly

Investigator's File is being kept in proper order

All monitoring visits must be documented. Any query must be discussed and resolved with the principal investigator.

8.11 Audit and Inspection

Auditors from SSI, regulatory authorities and ethics committees must be allowed access to all trial related documents, including the Investigator's File and the subjects' personal medical records.

8.12 Definition of source data

Source data is defined as the first documentation of any information related to the clinical trial which includes completed informed consent forms, medical records, laboratory results and hospital records.

8.13 Archiving

All trial related documentation, including source documentation, must be stored by the investigator until 2 years after the last marketing application for the product has been filed, or until SSI authorises the destruction of the documentation.

8.14 Agreement and financial settlement

All agreements between the Principal Investigators and SSI must be signed prior to inclusion of the first subject in the clinical trial. The agreement must clearly state the rights and obligations of the parties concerned and include a detailed financial settlement.

The trial is insured under SSI's product liability insurance under a worldwide liability programme. The policy covers claims arising from injury/injuries caused by trial medication used in clinical trials sponsored by the company, provided the trial medication has been used in accordance with the instructions given in the protocol.

9. Confidentiality and disclosure

All CRFs, information and results generated by Statens Serum Institut specific to H1/IC31®, patented or not, including patent applications and manufacturing processes not previously published, are considered confidential and shall remain the sole property of Statens Serum Institut.

An internal clinical trial report will be prepared in by Statens Serum Institut in co-operation with the Aurum Institute.

No data from the clinical trial may be published, presented or communicated, except to regulatory authorities, prior to the release of the internal clinical trial report, unless approved in writing by both Statens Serum Institut and The Aurum Institute in writing, which approval shall not be unreasonably withheld. The investigators agree not to discuss externally or publish any results from the trial without first submitting the results to Statens Serum Institut and allowing 30 days after receipt of the manuscript for the Statens Serum Institut to comment.

Concerning the publication, the names of the authors and their order of appearance will be discussed and consensus agreed upon by Senior Investigators at Aurum, Ifakara and SSI.

All intellectual property developed or derived from this study not pertaining specifically to the product will remain confidential between the parties. The parties agree that one party may not disclose any confidential information to any third party without the written consent of the other party, which such consent will not be unreasonably withheld.

All other confidentiality and intellectual property ownership matters pertaining to this protocol are set out in a separate agreement between the parties.

10. References

1. http://www.who.int/tb/publications/global-report/2006/en/.
2. Fine PEM. Variation in protection by BCG: implications of and for heterologous immunity. Lancet 1995; 346: 1339-45.
3. Doherty TM. New vaccines against tuberculosis. Tropical Medicine and International Health, 2004; 9(7): 818-26.
4. Brandt L. ESAT-6 subunit vaccination against Mycobacterium tuberculosis. Infect Immun, 2000; 68(2): 791-5.
5. Ravn P. Human T cell responses to the ESAT-6 antigen from Mycobacterium tuberculosis. J Infect Dis, 1999; 179:637-45.
6. Garcon N. et al. GlaxoSmithKline adjuvant systems in vaccines: concepts, achievements and perspectives. Expert Review. Vaccines 2007; 6(5), 723-739.
7. Lingnau K. Poly-L- arginine synergizes with oligodeoxynucleotides containing CpG-motifs (CpG-ODN) for enhanced and prolonged immune responses and prevents the CpG-ODN-induced systemic release of pro-inflammatory cytokines. Vaccine 20 (2002) 3498-3508.
8. van Dissel JT, Arend SM, Prins C, Bang P, Tingskov PN, Lingnau K, Nouta J, Klein MR, Rosenkrands I, Ottenhoff TH, Kromann I, Doherty TM, Andersen P. Ag85B-ESAT-6 adjuvanted with IC31 promotes strong and long-lived Mycobacterium tuberculosis specific T cell responses in naïve human volunteers. Vaccine. 2010 Apr 30;28(20):3571-81.
9. Agger EM, Rosenkrands I, Olsen AW, Hatch G, Williams A, Kritsch C, Lingnau K, von Gabain A, Andersen CS, Korsholm KS, Andersen P. Protective immunity to tuberculosis with Ag85B-ESAT-6 in a synthetic cationic adjuvant system IC31. Vaccine. 2006 Jun;24(26):5452-60.
10. van Dissel JT, Soonowala D, Joosten SA, Prinsa C, Arend SM, Bang P, Tingskov PN, Lingnau K, Nouta J, Hoff ST, Rosenkrands I, Kromann I, Ottenhoff THM, Doherty TM, Andersen P. Ag85B-ESAT-6 adjuvanted with IC31® promotes strong and long-lived Mycobacterium tuberculosis specific T cell responses in volunteers with previous BCG vaccination or tuberculosis infection. Vaccine (In Press).

11. Appendices

Appendix 1
Investigator's Statement

This clinical trial will be conducted in accordance with Good Clinical Practice (GCP), as defined by the European Community and the principles of the Declaration of Helsinki. In particular, the following points will be observed:

The final trial protocol and subsequent amendments will be complied with.

All subject data, including adverse events, will be completely and accurately recorded in the Case Report Forms (CRFs).

Serious Adverse Events and Adverse Drug Reactions, as defined in the protocol, will be reported to the appropriate regulatory authorities, ethics committees and to Statens Serum Institut, according to the instructions in the protocol.

The monitor will be allowed to review the subjects' medical records.

Before entering the trial, all of the participants must give their written informed consent.

The trial vaccines, properly stored, will only be supplied to subjects in the trial under the responsibility of the investigator/co-investigator. All used trial vaccine packages are accounted for in the vaccine dispensing log. Unused trial vaccines will be destructed at Statens Serum Institut, Denmark.

An Investigator's File, containing signed consent forms, screening log, correspondence and other information pertinent to the trial, will be kept at the investigator's/co-investigator's site during the recruitment/vaccination phase of the trial. The Investigator's File will be archived centrally by the investigator for 15 years after trial completion.

The Principal investigator will allow source data verification and audits by Statens Serum Institut and inspection by the health authorities.

I agree to the terms of this Statement and to Statens Serum Institut's general guidelines for conducting clinical trials.

Date: ____________________________

Name of Principal Investigator____Signature___________________

Appendix 2

Indemnity Statement

Trial title: Phase II Double-Blind, Randomized, Placebo-Controlled Study to Evaluate the Safety and Immunogenicity of H1/IC31®, an adjuvanted TB Subunit Vaccine, in HIV-Infected Adults with CD4+ Lymphocyte Counts Greater than 350 cells/mm^3

Dear ______________________

You have kindly agreed to consider undertaking the above-mentioned clinical trial of Statens Serum Institut's TB subunit vaccine as an investigator, in accordance with the protocol for the trial Aurum 102/THYB-05.

In the event that any recruited subject in the trial should suffer any personal injury resulting from the clinical trial, Statens Serum Institut agrees to indemnify the institution where the clinical trial is being undertaken (___________) and any of its employees or agents participating in the trial, against liability imposed by law, but not assumed voluntarily, and arising from the use of the TB subunit vaccine in the trial, provided that:

Statens Serum Institut shall not indemnify against, nor have any obligation whatsoever as regards liability arising from or related to any error, omission, intentional wrongful act, or other negligence on the part of said institutions or persons, such as medical malpractice; and

Any such institution or person seeking indemnity has fully complied with the trial protocol, and has promptly notified Statens Serum Institut of any notice of any type of claim, or the likelihood of a claim, relating to the trial, as regards any claim, makes no statement, takes no action, nor makes any commitment affecting Statens Serum Institut's interests, without Statens Serum Institut's prior written consent, and further, provides all reasonable and necessary assistance to Statens Serum Institut in the defence of any claim, allowing Statens Serum Institut, at its cost and in its discretion, to take over the defence of any action and to have full control in handling the claim.

Please note that this letter is not a legal contract itself, but rather summarizes the main points of Statens Serum Institut's liability under its agreement with ___________________.

Date: ________________________ Signature: ____

Ingrid Kromann
Department of Vaccine Development
Statens Serum Institut

Appendix 3

Division of Aids Table for Grading the Severity of
Adult and Pediatric Adverse Events, December, 2004
